# Impaired fatty acid oxidation and mitochondrial injury mediated by the AMPK/PPARα pathway: A key mechanism in high-fat diet-aggravated ulcerative colitis

**DOI:** 10.1371/journal.pone.0358354

**Published:** 2026-10-07

**Authors:** Huiyue Pan, Leilei Zhai, Shupeng Zhao, Ziyi Guo, Ping Yao

**Affiliations:** The First Department of Gastroenterology, The First Affiliated Hospital of Xinjiang Medical University, Urumqi, China; University of Kansas Medical Center, UNITED STATES OF AMERICA

## Abstract

**Background:**

A high-fat diet (HFD) is closely associated with the progression of ulcerative colitis (UC). However, the underlying mechanisms, particularly the interplay among lipid metabolic disturbances, mitochondrial damage, and intestinal barrier dysfunction, remain poorly defined. This study aimed to elucidate the molecular mechanisms by which HFD exacerbates DSS-induced UC, with a focus on the role of the AMPK/PPARα axis in mediating lipotoxicity and mitochondrial dysfunction that promote UC progression.

**Methods:**

A murine UC model was established in C57BL/6 mice by HFD feeding combined with DSS administration, along with an in vitro co-stimulation system using Caco-2 cells exposed to palmitic acid (PA) and lipopolysaccharide (LPS). Pathological phenotypes and molecular pathway alterations were systematically evaluated by hematoxylin and eosin (HE) staining, transmission electron microscopy (TEM), molecular biological techniques, and intervention with the specific AMPK agonist AICAR.

**Results:**

HFD significantly exacerbated DSS-induced colitis, as manifested by rapid weight loss, elevated disease activity index, and destruction of mucosal tissue architecture. Mechanistically, HFD led to intestinal epithelial lipid accumulation and gut microbiota dysbiosis, which synergistically triggered oxidative stress and paradoxically suppressed AMPK phosphorylation (p-AMPK). AMPK inactivation downregulates PPARα and its downstream fatty acid oxidation enzymes (CPT1A and ACOX1), exacerbating lipotoxicity and mitochondrial ultrastructural damage. Concurrently, decreased expression of key respiratory chain subunits (NDUFS2 and MTCO2) ultimately culminates in an energy crisis. Notably, pharmacological activation of AMPK using AICAR effectively reversed p-AMPK inhibition, restored PPARα expression, and ameliorated intestinal barrier function, demonstrating that the AMPK pathway serves as a critical pathogenic driver.

**Conclusion:**

HFD, through lipotoxicity and microbiota-derived pro-inflammatory signals, induces paradoxical inactivation of the intestinal epithelial AMPK/PPARα axis, which subsequently blocks fatty acid oxidation, provokes mitochondrial dysfunction and ATP depletion, and ultimately leads to breakdown of the intestinal barrier. This study uncovers a novel mechanistic link among metabolism, mitochondrial function, and gut barrier integrity, providing a solid theoretical and experimental foundation for precision metabolic reprogramming-based therapies in UC patients with metabolic derangements.

## Introduction

Ulcerative colitis (UC) is a form of inflammatory bowel disease (IBD) characterized by chronic inflammation and ulceration of the colonic mucosa, driven by aberrant inflammatory and immune responses [1]. The pathogenesis of UC remains incompletely understood and involves a complex interplay of genetic susceptibility, immune dysregulation, intestinal barrier dysfunction, and environmental factors such as diet [2]. In recent years, with the widespread adoption of Western-style diets, particularly HFD, the incidence of UC has been on the rise, suggesting that lipid metabolism disorders may play a significant role in the onset and progression of UC [3,4]. Notably, the incidence of UC in the Xinjiang region of China is relatively high compared to other areas, which may be attributed to the high-fat dietary habits of the local population [5]. Elucidating the mechanisms by which HFD contributes to UC pathogenesis is therefore of great clinical value for the development of targeted intervention strategies. However, systematic research is still lacking regarding the specific pathways by which HFD regulates host metabolism and interacts with the gut microbiota to drive the progression of UC, particularly mechanisms involving fatty acid oxidation (FAO) and mitochondrial function.

Excessive lipid intake can overload lipid metabolism in intestinal epithelial cells, leading to impaired FAO [6]. When the FAO capacity of intestinal epithelial cells is compromised, lipids accumulate within the cells and trigger lipotoxicity. As the primary site of fatty acid β-oxidation, the functional state of mitochondria directly influences lipid utilization efficiency and cellular energy homeostasis [7,8]. Furthermore, as a key nuclear receptor that governs this lipid metabolic network, peroxisome proliferator-activated receptor α (PPARα) largely determines the efficiency of mitochondrial FAO through its expression activity. Dysregulated lipid metabolism induces mitochondrial ultrastructural damage and functional impairment. Subsequently, mitochondria face an energy crisis due to disruption of the electron transport chain, manifested as insufficient ATP production and the accumulation of reactive oxygen species (ROS) [9]. This energy deficiency and oxidative stress, in conjunction with the effects of lipotoxic molecules on junctional complexes, lead to a loss of tight junction protein expression, disrupt the intestinal barrier, and consequently exacerbate the intestinal inflammatory response [10].

As the second genome of host metabolism and immunity, the gut microbiota has a structure and function that are highly susceptible to dietary intervention. HFD consumption significantly reduces microbial α-diversity, promotes the enrichment of pathogenic bacteria, and suppresses beneficial commensals, thereby impairing the mucus layer barrier function and disrupting immune tolerance [11,12]. Dysbiotic microbiota can influence energy sensing and metabolic signaling pathways in intestinal epithelial cells through the modulation of metabolites such as short-chain fatty acids (SCFAs) [13]. Consequently, gut microbiota dysbiosis may disrupt the homeostatic balance between fatty acid oxidation and mitochondrial function by interfering with fatty acid metabolic pathways, thereby driving intestinal epithelial lipotoxicity and energetic crisis. Therefore, modulating the gut microbiota and the fatty acid metabolic axis to restore fatty acid oxidation and mitochondrial homeostasis may represent a promising therapeutic strategy for UC [14,15].

AMP-activated protein kinase (AMPK) functions as a master sensor of cellular energy metabolism and plays a central role in regulating fatty acid oxidation, mitochondrial biogenesis, and oxidative stress responses [16–18]. PPARα is a key nuclear receptor that regulates the expression of genes involved in fatty acid oxidation; its transcriptional activity is primarily governed by AMPK-mediated phosphorylation [19]. Inhibition of AMPK suppresses the expression of CPT1A, the rate-limiting enzyme of mitochondrial fatty acid oxidation, and ACOX1, a critical enzyme of peroxisomal fatty acid oxidation—both of which are downstream targets of PPARα—thereby exacerbating lipid metabolic disturbances and mitochondrial dysfunction [20]. Crucially, recent transcriptomic profiling based on clinical UC cohorts has revealed significant impairments in mucosal fatty acid β-oxidation, with the suppression of the PPAR signaling pathway emerging as a core molecular event. Protein-protein interaction (PPI) network analyses further highlighted that PPAR-regulated key metabolic enzymes act as highly connected hubs in the pathogenesis of UC [21]. Previous studies and our preliminary research have confirmed that the PPARα signalling pathway is markedly suppressed in ulcerative colitis (UC) models, and that restoring its activity ameliorates intestinal inflammation and lipid abnormalities [22,23]. Although the AMPK/PPARα axis has been extensively investigated in metabolic disorders such as non-alcoholic fatty liver disease (NAFLD), its causal role in bridging diet-induced lipid dysregulation with intestinal barrier dysfunction in UC remains elusive [24]. Based on current evidence, we propose the following hypothesis: the combined action of HFD-induced lipotoxicity and gut microbiota dysbiosis-mediated inflammation triggers a robust burst of intracellular ROS. Although AMPK normally acts as a protective factor in response to metabolic stress, this excessive oxidative stress, via modification of its upstream kinase activity. Paradoxically, it leads to AMPK inactivation. This in turn interferes with PPARα-mediated transcription of key fatty acid oxidation enzymes. The consequent metabolic defects ultimately result in mitochondrial structural damage, an ATP energy crisis, and a pronounced loss of tight junction proteins, thereby compromising the intestinal physical barrier function.

To systematically test this hypothesis, we established a mouse colitis model induced by DSS after 8 weeks of HFD feeding and analyzed host–microbiome interactions via 16S rRNA sequencing and microbial metabolic functional prediction. In combination with in vitro models and specific pharmacological interventions, we obtained relatively robust causal evidence. Our findings elucidated the mechanistic roles of metabolism, gut microbiota, and inflammation in the pathogenesis of ulcerative colitis, thereby laying a theoretical foundation for precision therapies that target metabolic reprogramming to restore mitochondrial function.

## Materials and methods

### Reagents and materials

Palmitic acid (HY-N0830) was purchased from MedChemExpress (MCE, Monmouth Junction, NJ, USA). Lipopolysaccharide (L2880) was purchased from Sigma-Aldrich (St. Louis, MO, USA). AICAR (HY-13417) was purchased from MedChemExpress (MCE, Monmouth Junction, NJ, USA). Compound C (HY-13418) was purchased from MedChemExpress (MCE, Monmouth Junction, NJ, USA). Acetylcysteine (NAC) (HY-B0215) was purchased from MedChemExpress (MCE, Monmouth Junction, NJ, USA). Primary antibodies against AMPK (A27740), phospho-AMPK (AP0871), ACOX1 (A22366), CPT1A (A27657), Occludin (A2601), and GAPDH (A19056) were purchased from ABclonal Technology (Woburn, MA, USA). Anti-PPARα antibody (bs-233398R) was purchased from Bioss Antibodies (Beijing, China). Anti-Claudin1 antibody (AF0127) was purchased from Affinity Biosciences (Cincinnati, OH, USA). Antibodies against NDUFS2 (M05618-1) and MTCO2 (MA03631-1) were purchased from Boster Biological Technology (Pleasanton, CA, USA). All secondary antibodies (AS014) were purchased from ABclonal Technology (Woburn, MA, USA). Protease and phosphatase inhibitor (Cat. #GRF103), RIPA lysis buffer (Cat. #PC101), BCA protein assay kit (Cat. #ZJ102), and 10% polyacrylamide gel electrophoresis kit (Cat. #PG112) were all purchased from EpiZyme, Shanghai. Polyvinylidene fluoride (PVDF) membrane (Cat. #IPVH00010) was purchased from Millipore, USA. Caco-2 cells were obtained from Procell Life Science & Technology Co., Ltd. (Wuhan, China) and were authenticated by short tandem repeat (STR) profiling

### Animals and treatments

Male C57BL/6 mice (4 weeks old, weighing 10–15 g) were obtained from the Animal Experiment Center of Xinjiang Medical University (Production/Use License No.: SYXK (Xinjiang) 2018-0003). All animals were housed under a 12 h light/dark cycle with free access to food and water. The ambient temperature was maintained at 21–26°C with a relative humidity of 40%–70%. All experimental procedures were approved by the Animal Experiment Medical Ethics Committee of the First Affiliated Hospital of Xinjiang Medical University (Ethics Approval No.: IACUC-JT-20231227-14). After one week of acclimatization, mice were randomly assigned to either a normal diet group or a HFD group and maintained on the respective diets for 8 weeks. At the end of the 8-week feeding period, mice fed the normal diet were further subdivided into a control group (CH) and an UC model group (CD). Mice fed the HFD were subdivided into a high-fat diet control group (HH) and a high-fat diet plus UC model group (HD). To induce UC, mice in the CD and HD groups were given 3% DSS solution ad libitum for 7 days, while mice in the CH and HH groups continued to receive normal drinking water. At the end of the experiment, all mice were anesthetized with isoflurane inhalation and then euthanized by cervical dislocation. To alleviate animal suffering, the bedding thickness was increased, and cages were cleaned 2–3 times per day to keep them dry and clean, preventing perianal skin ulceration and secondary infections. Before euthanasia, blood was collected from all mice via the retro-orbital venous sinus (performed under anesthesia to ensure a painless procedure). A portion of the colon tissue was collected and fixed in 4% paraformaldehyde. The remaining fresh colon tissue was snap-frozen in liquid nitrogen and stored at −80°C until further analysis.

### 16S rRNA gene sequencing and data analysis

16S rRNA gene sequencing was performed by Metware Biotechnology Co., Ltd. (Wuhan, China). The raw sequencing data have been deposited into Figshare (https://doi.org/10.6084/m9.figshare.32901530). The V4 hypervariable region of the 16S rDNA gene was amplified by PCR, and the amplicons were sequenced on the Illumina NovaSeq PE250 platform to characterize the composition of the gut microbiota. Raw sequencing reads were subjected to quality filtering to obtain clean data, followed by denoising using the Deblur algorithm to generate amplicon sequence variants (ASVs). Taxonomic annotation of the ASVs was then performed. For diversity analyses, both alpha and beta diversity were assessed based on ASV abundance. Alpha diversity indices, including the Shannon and Chao1 indices, were calculated using the phyloseq and vegan packages in R software to evaluate within-sample species richness and evenness. For beta diversity analysis, the Bray-Curtis distance was calculated using the phyloseq package, and principal coordinate analysis (PCoA) plots were generated with the ggplot2 package to visualize differences in microbial community structure among samples. Linear discriminant analysis effect size (LEfSe) was employed to identify differentially enriched taxa between groups. Finally, the functional profile of the microbial community was predicted using PICRUSt2 based on the KEGG database.

### Hematoxylin and eosin (H&E) staining

Colon tissue samples were fixed in 4% paraformaldehyde, embedded in paraffin, and sectioned at a thickness of 4 μm. The sections were then stained with H&E. Stained sections were examined under a light microscope for histopathological evaluation.

### Enzyme-linked immunosorbent assay (ELISA)

Serum samples were obtained by centrifuging blood collected from the eyeballs of mice. The levels of IL-1β, IL-6, TNF-α, and IFN-γ were measured using ELISA kits (Shanghai Jianglai Biotechnology Co., Ltd., Shanghai, China) according to the manufacturer’s instructions. The absorbance (optical density, OD) of each well was measured at 450 nm.

### Biochemical analysis

The levels of total triglyceride (TG), total cholesterol (TC), and low-density lipoprotein cholesterol (LDL-C) in plasma were measured using commercial assay kits (Elabscience Biotechnology Co., Ltd., Wuhan, China) according to the manufacturer’s instructions. Serum samples from different groups were assessed, and the OD values were measured at 510 nm for TG and TC, and at 546 nm for LDL-C.

### Transmission electron microscopy (TEM)

Fresh colon tissue was cut into 1 mm³ pieces and immediately fixed with 1% osmium tetroxide. After dehydration through a graded ethanol series, the specimens were embedded in acetone and 812 embedding medium, and 60 nm sections were cut using an ultramicrotome. The sections were double-stained with uranyl acetate and lead citrate, and then observed under a transmission electron microscope (model: HT7800) to examine the ultrastructure of intestinal epithelial cells and mitochondria.

### Measurement of adenosine triphosphate (ATP) levels

ATP levels in mouse colon tissues were measured using a commercial assay kit (Elabscience Biotechnology Co., Ltd., Wuhan, China) according to the manufacturer’s instructions. The ATP concentration was determined by measuring the OD at 636 nm.

### Western blotting

Total protein was extracted from frozen colon tissues using RIPA lysis buffer supplemented with a protease and phosphatase inhibitor cocktail. Protein concentrations were determined using a BCA protein assay kit according to the manufacturer’s instructions. Equal amounts of protein were separated by sodium dodecyl sulfate-polyacrylamide gel electrophoresis (SDS-PAGE) using 10% polyacrylamide gel electrophoresis kit. The separated proteins were then transferred onto PVDF membranes. Membranes were blocked with 5% nonfat milk in TBST for 1.5 h at room temperature to prevent non-specific antibody binding. Subsequently, the membrane was incubated with the primary antibody at an appropriate dilution overnight at 4°C. The following primary antibodies and dilutions were used: AMPK (1:2000), phospho-AMPK (1:1000), PPARα (1:500), ACOX1 (1:2000), CPT1A (1:1000), Occludin (1:2000), Claudin-1 (1:1000), NDUFS2 (1:1000), MTCO2 (1:1000), and GAPDH (1:5000). After washing with TBST, membranes were incubated with horseradish peroxidase (HRP)-conjugated secondary antibody (1:10000) for 1 h at room temperature. Protein bands were visualized using an Omni-ECL enhanced chemiluminescence kit (SQ101; EpiZyme, Shanghai, China) and imaged with an eBlot Touch imaging system (eBlot, Shanghai, China). Densitometric analysis was performed using ImageJ software (National Institutes of Health, Bethesda, MD, USA), and relative protein expression levels were normalized to GAPDH as an internal reference. Each Western blot experiment was repeated at least three times to ensure reproducibility.

### Cell culture and treatment

Caco-2 cells were cultured in a humidified atmosphere containing 5% CO2 at 37°C. The culture medium was supplemented with 20% fetal bovine serum (FBS), penicillin (100 U/mL), and streptomycin (100 μg/mL). Cell viability was assessed using the Cell Counting Kit-8 (CCK-8) assay to evaluate the cytotoxicity of AICAR, Compound C, NAC, LPS, and PA. Caco-2 cells were seeded in 96-well plates and cultured for 24 h. Subsequently, cells were treated with various concentrations of each reagent, with three replicate wells per group. After 24 h of exposure to the reagents, cells were incubated with 10% CCK-8 solution for 2 h. At 450 nm, the OD was calculated using a microplate reader (Thermo Scientific, Waltham, United States).

### Oil Red O staining

Oil Red O staining of cells was performed using an Oil Red O staining kit (Solarbio Science & Technology Co., Ltd., Beijing, China), following the manufacturer’s instructions. Briefly, Caco-2 cells were washed twice with phosphate-buffered saline (PBS) and fixed with Oil Red O fixative for 20 min. After a rapid rinse with 60% isopropanol, the cells were incubated with freshly prepared Oil Red O working solution for 15 min. Excess stain was removed by differentiation with 60% isopropanol followed by two washes with distilled water. Stained samples were examined under a light microscope, and the staining intensity was quantified using ImageJ software.

### Detection of ROS

ROS levels were measured using a ROS Assay Kit (CA1410; Solarbio Science & Technology Co., Ltd., Beijing, China) according to the manufacturer’s instructions. Briefly, after treatment, the culture medium was removed, and cells were washed twice with PBS. Cells were then incubated with 5 μmol/L of the fluorescent probe 2′,7′-dichlorodihydrofluorescein diacetate (DCFH-DA) at 37°C for 30 min. Fluorescence was visualized under a fluorescence microscope with excitation/emission wavelengths of 488 nm and 525 nm, respectively. Images were captured and quantified using ImageJ software.

### Analysis of mitochondrial membrane potential (MMP)

MMP was assessed using a JC-1 Assay Kit (M8650; Solarbio Science & Technology Co., Ltd., Beijing, China) according to the manufacturer’s instructions. MMP was detected using the mitochondrial-specific fluorescent probe JC-1. Briefly, cells were incubated with JC-1 staining working solution at 37°C for 20 min, followed by washing with JC-1 staining buffer. Fluorescence signals were observed under a fluorescence microscope, with JC-1 monomers detected at excitation/emission wavelengths of 490/530 nm and JC-1 aggregates at excitation/emission wavelengths of 525/590 nm. The fluorescence intensity was quantified using ImageJ software.

### Statistical analysis

Statistical analysis was performed using GraphPad Prism 10.4.2 software (GraphPad Software, Inc., San Diego, CA, USA; https://www.graphpad-prism.cn). Data are presented as the mean ± standard deviation (SD) from at least three independent experiments. Comparisons between two groups were analyzed using the Student’s *t*-test, while comparisons among multiple groups were assessed using one-way analysis of variance (ANOVA) followed by the appropriate post hoc test. All in vivo and in vitro experiments were conducted with randomization. A P value < 0.05 was considered statistically significant.

## Results

### HFD exacerbates DSS-induced colitis symptoms and intestinal damage

To explore how different dietary patterns affect systemic and intestinal inflammation, we first placed mice on an 8-week dietary intervention. During this phase, body weight changes were monitored, revealing significant differences between the normal diet group and the high-fat diet group (Fig 1A). Following the 8-week period, colitis was induced over 7 days. As expected, mice in both the CD and HD groups exhibited typical clinical symptoms of colitis, including continuous body weight loss and elevated Disease Activity Index (DAI) scores. Notably, compared with the CD group, the HD group displayed significantly exacerbated disease progression, characterized by a notably accelerated trajectory of body weight loss (Fig 1B) and drastically augmented DAI scores overall (Fig 1C). Colon length is a well-recognized macroscopic marker of gut inflammation. In our experiment, it was significantly reduced in the CD and HD groups compared with their corresponding healthy control groups (CH and HH). Interestingly, even among healthy mice, the HH group had shorter colons than the CH group. In the disease groups, colon shortening was more pronounced in the HD group than in the CD group (Fig 1E). Histopathological examination of colon sections stained with H&E further supported these observations. The CH group displayed intact mucosa, regularly aligned crypts, and no obvious inflammatory infiltration. The HH group showed largely normal architecture. In contrast, the CD group exhibited classic inflammatory changes, such as goblet cell depletion, partial crypt disruption, and immune cell infiltration. The most severe damage occurred in the HD group, which featured extensive mucosal ulceration, severe crypt loss or complete destruction, and dense inflammatory cell accumulation (Fig 1D). Overall, we conclude that HFD exacerbates colonic inflammation brought on by DSS.

**Fig 1:**
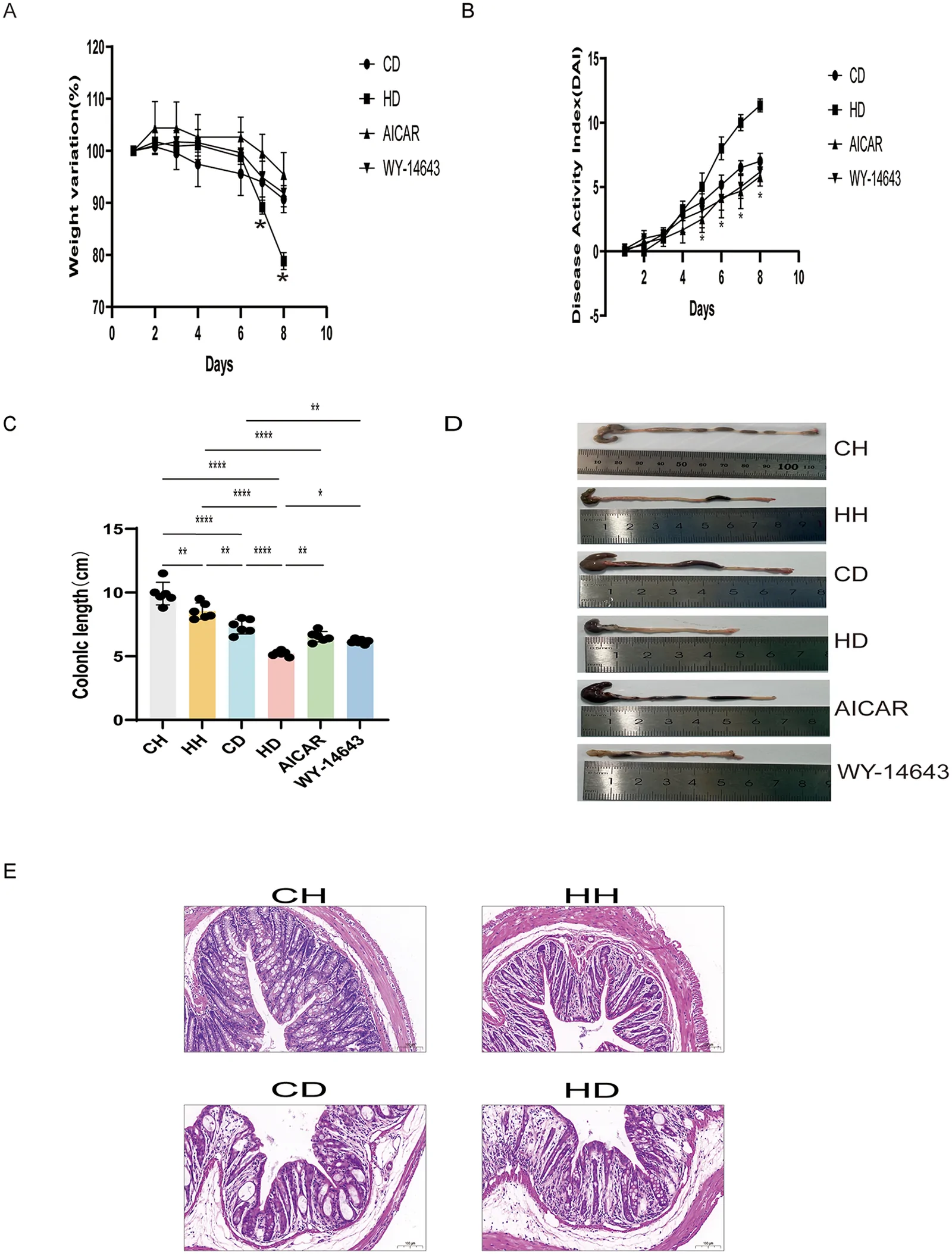
(A) Body weight variation (%) of mice during the 8-week (normal diet (CH and CD) or high-fat diet (HH and HD)) period (n = 6 per group). **** p < 0.0001 (B-C) Body weight loss percentage (B) and Disease Activity Index (DAI) (C) over the 7-day (DSS administration) period, **** p < 0.0001. (D) Representative H&E staining images of colonic sections from the CH, HH, CD, and HD groups. Scale bar: 200 μm. (E) Comparison of colon length in DSS-induced colitis mice fed a normal diet or a high-fat diet, with representative images of colon specimens. Data are presented as mean ± standard deviation (SD). *p < 0.05, ****p < 0.0001.

### HFD induces lipid metabolic disorders and further amplifies the inflammatory response in UC

To evaluate the effects of HFD and UC on systemic lipid metabolism and inflammatory status, we measured serum lipoprotein and pro-inflammatory cytokine levels in mice from each group. HFD significantly upregulated lipid metabolism-related indicators. Quantitative analysis of TC, TG, and LDL-C revealed that HFD markedly altered the serum lipid profile of mice. Compared with the normal diet group, the levels of TC, TG, and LDL-C in the HFD group were significantly increased, suggesting that the high-fat diet induced lipid metabolism disorders (Fig 2A). Compared with the CH group, the HH group showed a slight increase in the levels of TNF-α and IFN-γ. The levels of various inflammatory factors in the CD group were significantly higher than those in the CH and HH groups. Compared with the CD group, the HD group exhibited a more significant increase in inflammatory factor levels (Fig 2B). This finding suggests that HFD-associated metabolic abnormalities are closely linked to a low-grade chronic inflammatory state and correlate with an exacerbated release of pro-inflammatory cytokines during colitis.

**Fig 2:**
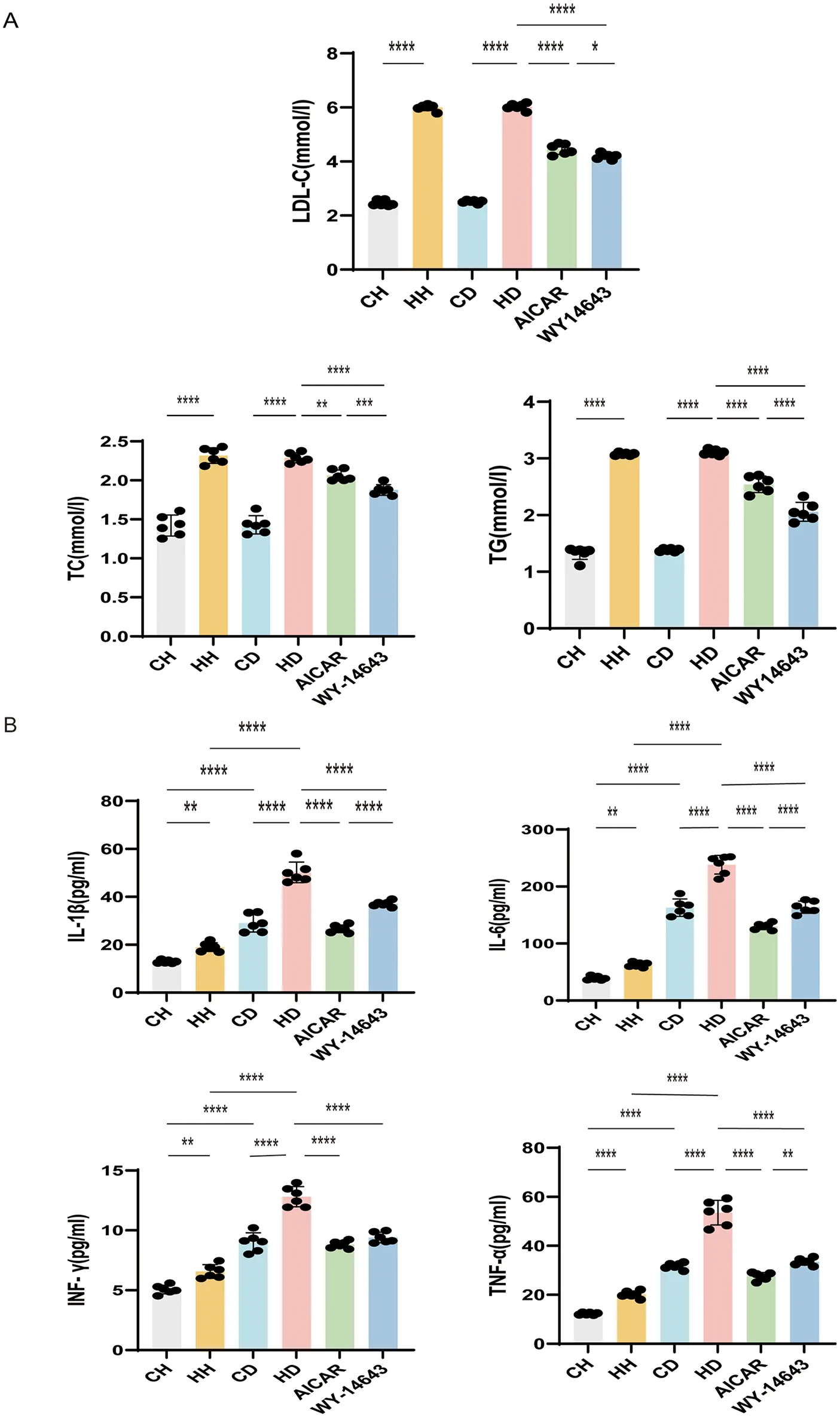
(A) Quantitative analysis of serum lipid metabolism markers in mice from each group, including LDL-C, TC, and TG. (B) Expression levels of pro-inflammatory cytokines in serum of mice from each group, including IL-1β, IL-6, TNF-α, and IFN-γ. Data are presented as the mean ± standard deviation (SD). *p < 0.05, **p < 0.01, ***p < 0.001, ****p < 0.0001.

### HFD-induced gut microbiota dysbiosis is associated with altered microbial metabolic functions in experimental colitis

To investigate the effects of dietary intervention and UC on the intestinal microbiota, we performed 16S rRNA gene sequencing analysis. First, species composition analysis at the phylum level revealed a reorganization of the microbial community structure (Fig 3A). Firmicutes were predominant in the CH and CD groups but decreased following HFD intervention. In the HD group, Proteobacteria and Verrucomicrobiota were markedly increased. Alpha diversity analysis indicated that HFD significantly reduced the abundance and diversity of the gut microbiota. Compared with the normal groups (CH and CD), the HFD groups (HH and HD) exhibited decreased Shannon indices (Fig 3B) and Chao1 indices (Fig 3C), with the HD group showing the lowest diversity. Subsequently, Principal Coordinate Analysis (PCoA) demonstrated statistically distinct clustering among the four groups, indicating macroscopic structural shifts in the microbial communities driven independently and synergistically by diet and DSS treatment (Fig 3D). To further identify key differentially abundant biomarkers, we performed LEfSe analysis between the CD and HD groups (Fig 3E). The results showed that the CD group was enriched with numerous beneficial commensal bacteria, such as Clostridia and Lachnospiraceae under the phylum Firmicutes. In contrast, the HD group was highly enriched with potentially pathogenic taxa, including Proteobacteria and Campylobacteria. Next, we utilized PICRUSt2 analysis to infer the functional potential of these altered microbial communities (Fig 3F). The prediction revealed significant functional shifts in the gut microbiome of the HD group compared to the CD group, characterized by widespread alterations in bacterial metabolic modules. Collectively, these observational data suggest that HFD-induced dysbiosis shapes a disrupted luminal metabolic microenvironment. Rather than exerting a direct causal regulation on specific host eukaryotic pathways, this microbial alteration serves as an indispensable pro-inflammatory context that is highly associated with the host’s worsened inflammatory phenotype during exogenous DSS challenge.

**Fig 3:**
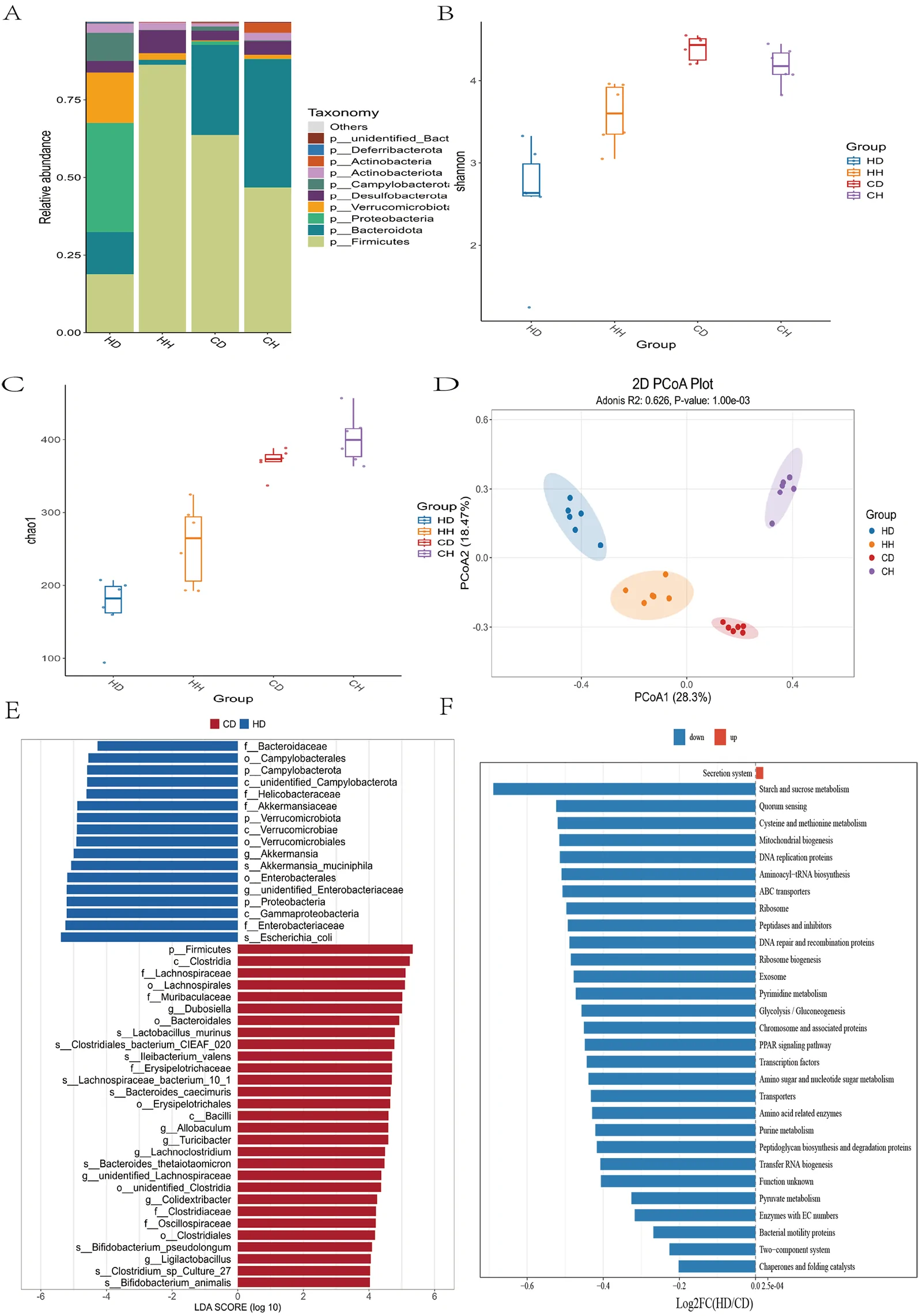
(A) Bar plot showing the relative abundance of gut microbiota at the phylum level in each group of mice. (B–C) Alpha diversity analysis of the gut microbiota in mice, including (B) Shannon index and (C) the Chao1 index, presented as box-and-whisker plots. *p < 0.05, **p < 0.01, ***p < 0.001. (D) Beta diversity analysis of the gut microbiota in mice, shown as a PCoA scatter plot. (E) LEfSe analysis of differentially abundant taxa between the CD and HD groups. (F) Functional pathway prediction analysis based on microbial abundance between the CD and HD groups.

### HFD feeding exacerbates DSS-induced impairment of the colonic AMPK/PPARα metabolic axis and epithelial barrier integrity in vivo

To further investigate the potential molecular mechanisms by which a HFD exacerbates UC, Western blotting was employed to evaluate the expression of proteins related to FAO signaling pathways, the mitochondrial oxidative respiratory chain, and tight junctions in the colon tissues of each group (Fig 4). The results indicated that the suppression of the AMPK-PPARα signaling pathway and FAO was highly correlated with HFD exposure under inflammatory conditions. Compared with the CH group, we observed a decrease in the phosphorylation activation of AMPK (p-AMPK/AMPK ratio) and the protein expression of the downstream core metabolic transcription factor PPARα in both the HH and CD groups. Furthermore, the downregulation of AMPK phosphorylation and PPARα expression was even more pronounced in the HD group. Coinciding with this suppressed upstream signaling, the protein expression levels of CPT1A and ACOX1, which are key rate-limiting enzymes of fatty acid oxidation regulated by PPARα, were correspondingly downregulated. This indicates that the HFD-associated metabolic shift closely aligns with abnormal lipid oxidative metabolism in intestinal cells under disease conditions. Given that fatty acid oxidation depends on mitochondrial integrity and function, we assessed the core subunits of the mitochondrial respiratory chain, NDUFS2 (complex I) and MTCO2 (complex IV). The data demonstrated that the HFD environment was accompanied by more severe mitochondrial damage under DSS induction, as evidenced by the significant downregulation of NDUFS2 and MTCO2 protein expression in the HD group. Crucially, we found a strong correlation between these metabolic/mitochondrial alterations and the loss of physical barrier proteins. As marker molecules of the intestinal epithelial mechanical barrier, the protein expressions of Occludin and Claudin-1 were significantly downregulated in the HD group. In summary, these in vivo observations demonstrate that HFD exposure is intrinsically linked to a suppressed AMPK-PPARα signaling axis, impaired fatty acid oxidation, and disrupted mitochondrial homeostasis. Furthermore, this broad metabolic dysfunction coincides tightly with the loss of required energy support and the severe depletion of key tight junction proteins, exacerbating the development and progression of UC.

**Fig 4:**
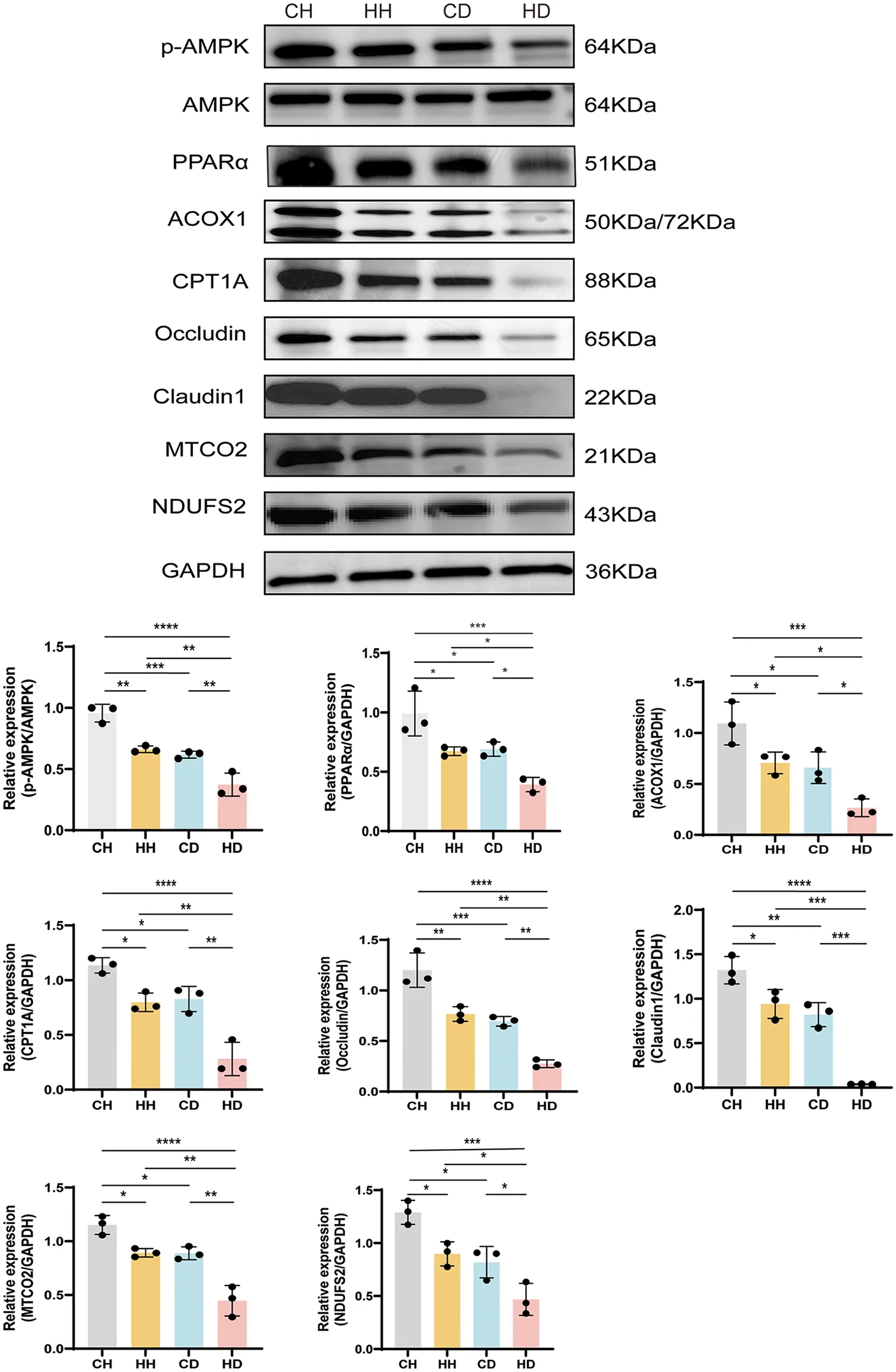
Representative Western blot images showing protein expression levels of p-AMPK, AMPK, PPARα, ACOX1, CPT1A, MTCO2, NDUFS2, Occludin, and Claudin1 in colon tissues of mice from each group. GAPDH was used as a loading control for normalization (n = 3). Quantitative analysis of protein expression levels in each group. Data are presented as the mean ± standard deviation (SD). *p < 0.05, **p < 0.01, ***p < 0.001, ****p < 0.0001.

### HFD exacerbates DSS-induced intestinal ultrastructural damage and energy metabolism disorders

To evaluate how HFD affects the ultrastructure of intestinal epithelial cells, we examined colon tissues from each group under a TEM (Fig 5A). In the CH group, microvilli were dense, well organized, and structurally intact. Mitochondria exhibited normal oval or elongated morphologies with continuous double membranes and densely packed cristae. In the HH group, the overall ultrastructure remained largely normal, though there was a slight decrease in electron density within the mitochondrial matrix and a modest widening of the cristae spaces. The CD group showed clear pathological damage: the microvilli became noticeably shorter, sparser, and unevenly arranged. Their mitochondria were swollen and spherical, with disrupted and fewer cristae, and we observed electron-dense granules in the matrix. The HD group exhibited the most severe damage, characterized by extensive loss or shedding of microvilli. Mitochondria in the HD group were severely swollen and displayed widespread vacuolization, ruptured double membranes, and complete loss of cristae. Therefore, given that these marked ultrastructural alterations strongly suggested impaired energy metabolism, we subsequently quantified ATP levels in colonic tissues (Fig 5B). The CH group maintained relatively high ATP levels. Both the HH and CD groups had lower ATP levels than the CH group. The HD group showed the most notable reduction in ATP content, and the difference from the CD group was statistically significant. Collectively, these results indicate that HFD induces mitochondrial structural damage, leading to energetic collapse, thereby disrupting intestinal epithelial integrity and exacerbating DSS-induced colitis.

**Fig 5:**
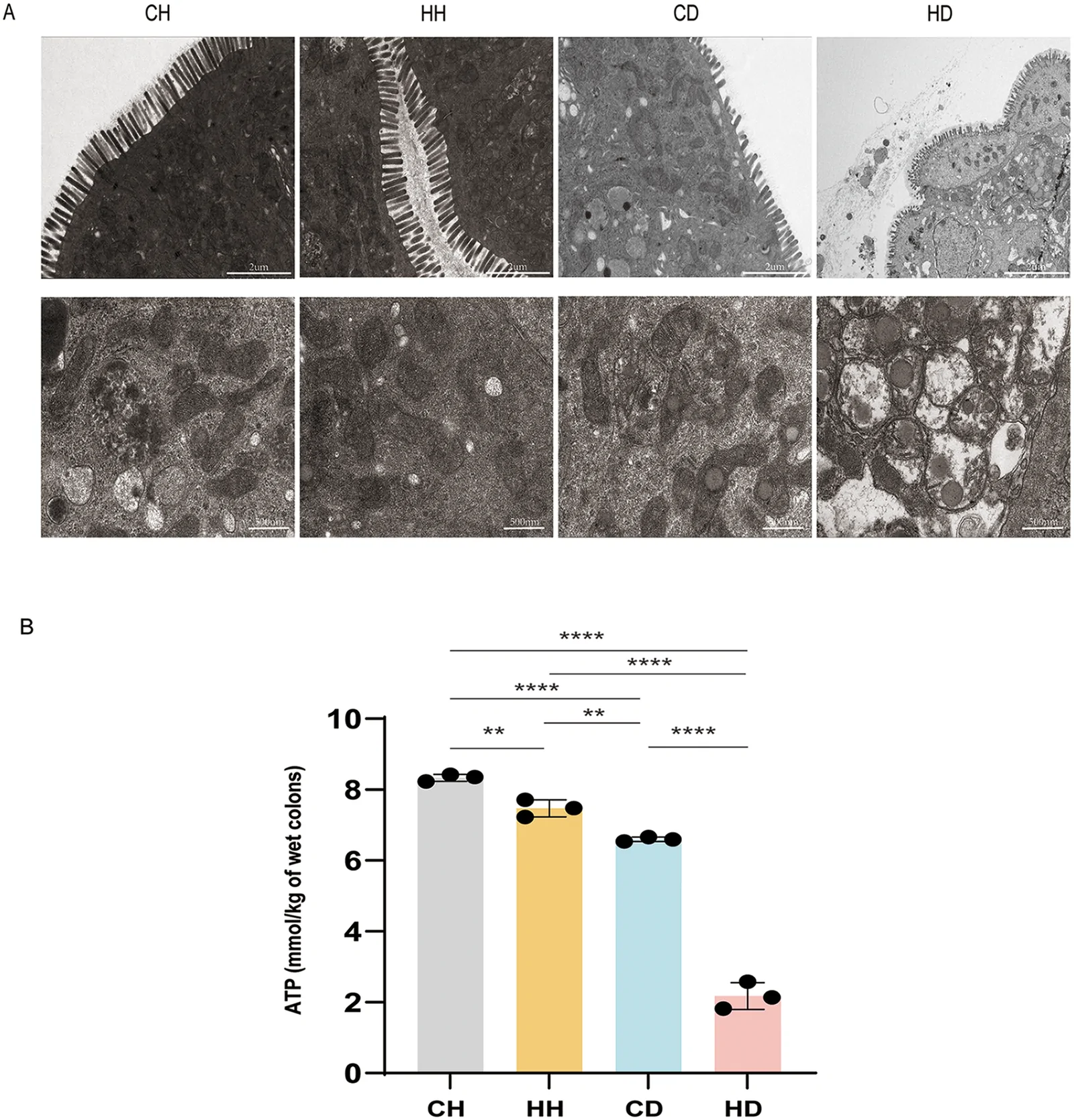
(A) TEM images showing ultrastructural changes in colonic epithelial cells of mice from each group. Scale bars: 2 μm and 500 nm. (B) Quantitative analysis of ATP content in colon tissues of mice from each group. Data are representative of three independent experiments. **p < 0.01, ****p < 0.0001.

### Assessment of cytotoxicity and establishment of an in vitro lipotoxicity model in Caco-2 cells

To investigate the molecular mechanisms by which a HFD exacerbates colitis, we performed in vitro experiments using human colorectal adenocarcinoma Caco-2 cells. To exclude potential confounding effects from severe cell death on downstream signaling analyses, we evaluated cell viability using the CCK-8 assay across a range of concentrations of reagents (Fig 6A), including the reactive oxygen species (ROS) scavenger N-acetylcysteine (NAC), the AMPK inhibitor Compound C, the AMPK activator AICAR, LPS and PA. Based on these results, we selected safe concentration ranges that did not significantly compromise Caco-2 cell viability for subsequent interventions (i.e., 5 mM NAC, 0.5 mM AICAR, 10 μM Compound C, 1 μg/mL LPS, and 0.4 mM PA). To evaluate the effects of PA on lipid metabolism in Caco-2 cells and to establish an in vitro lipid accumulation model, we treated Caco-2 cells with PA and performed morphological observation and quantitative analysis of lipid deposition. Microscopic examination revealed that, compared with the control group, the PA-treated group exhibited a large number of lipid droplets within Caco-2 cells. Quantitative analysis of the relative lipid droplet area further confirmed that the relative lipid droplet area in the PA group was significantly higher than that in the NC group, with statistically significant differences (Fig 6B). These results demonstrate that PA treatment induces significant lipid accumulation in Caco-2 cells.

**Fig 6:**
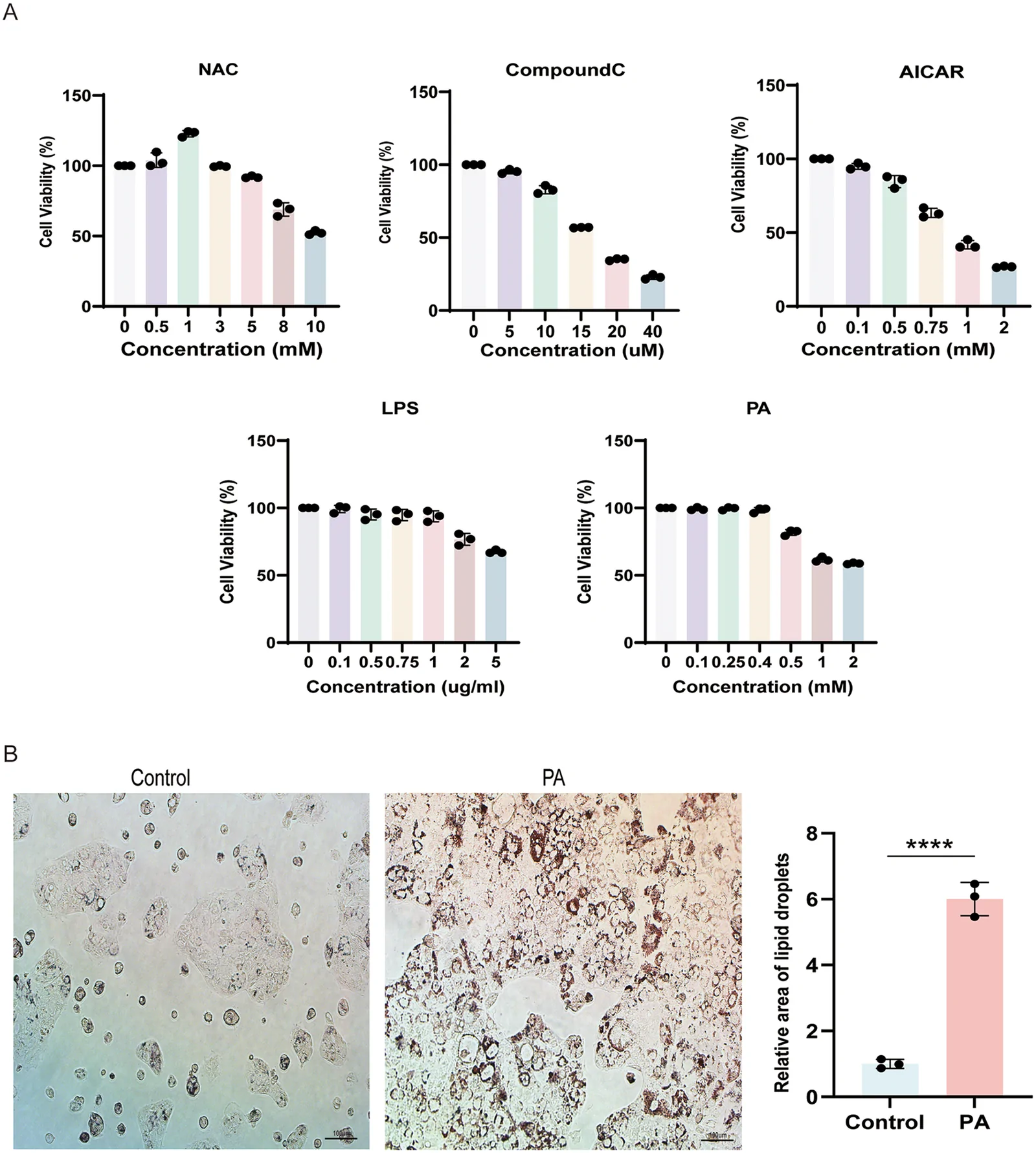
(A) Effects of NAC, Compound C, AICAR, LPS and PA on the viability of Caco-2 cells. (B) Effect of PA on intracellular lipid accumulation. Scale bar: 100 μm. ****p < 0.0001. Data are representative of three independent experiments.

### Lipotoxic and inflammatory stress synergistically disrupt the AMPK/PPARα axis and tight junctions, which can be rescued by AMPK activation

To mimic the in vivo pathological microenvironment of HFD-exacerbated colitis at the cellular level, we challenged Caco-2 intestinal epithelial cells with PA and LPS. Western blot analysis (Fig 7) revealed that while treatment with either PA or LPS alone induced a reduction in the p-AMPK, the co-treatment (LPS + PA) synergistically suppressed AMPK activation. Concordantly, the expression of PPARα and its downstream fatty acid oxidation targets (ACOX1, CPT1A) were drastically downregulated in the co-treatment group. This lipid metabolic disorder was accompanied by mitochondrial dysfunction, as evidenced by the downregulation of the mitochondrial respiratory chain subunits MTCO2 and NDUFS2. Concurrently, this metabolic aberration was tightly mirrored by impaired epithelial barrier integrity, manifested by decreased expression of the tight junction proteins Occludin and Claudin‑1.

**Fig 7:**
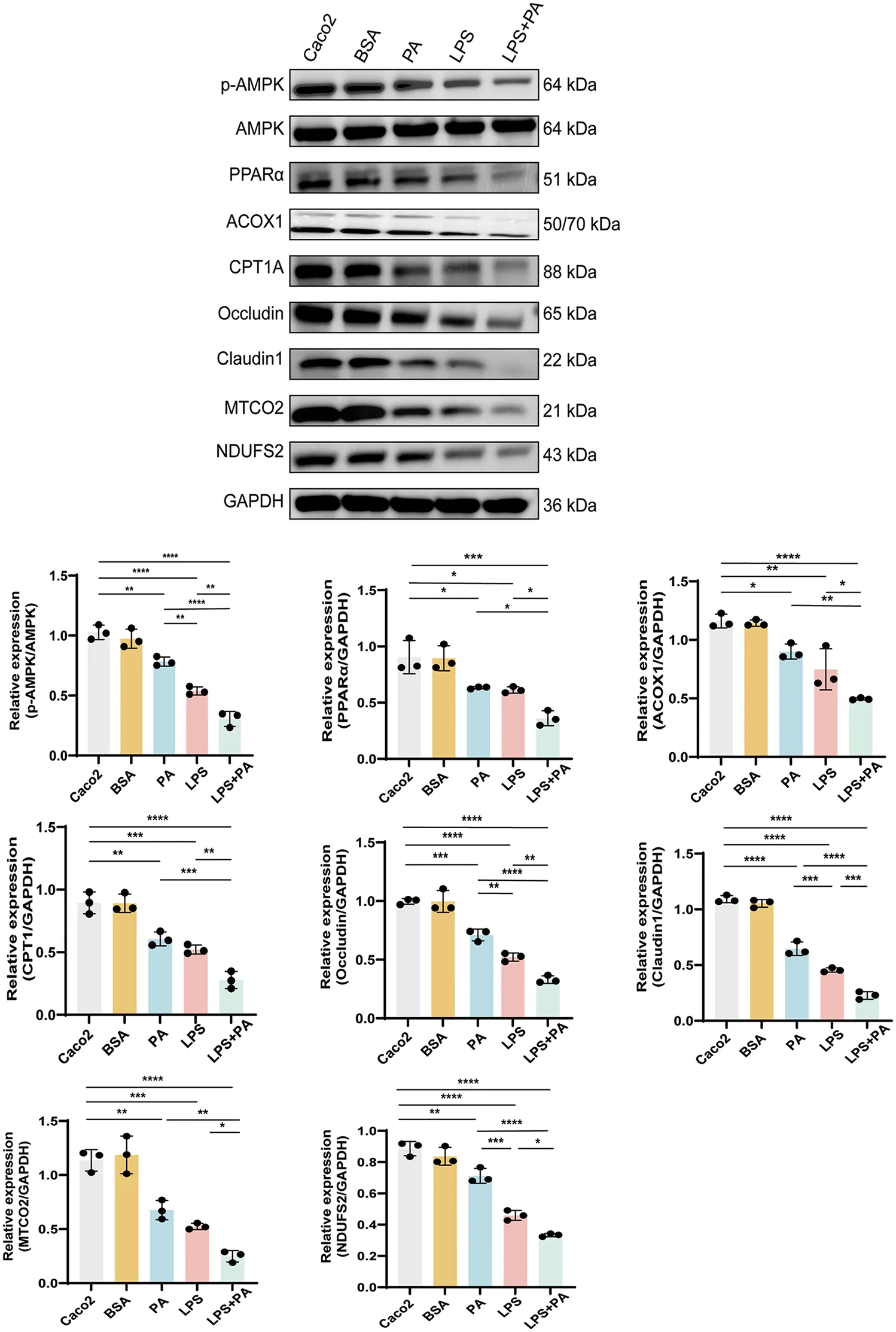
Representative Western blot images showing protein expression levels of p-AMPK, AMPK, PPARα, ACOX1, CPT1A, MTCO2, NDUFS2, Occludin, and Claudin1 in Caco-2 cells from each group. GAPDH was used as a loading control for normalization (n = 3). Quantitative analysis of protein expression levels in each group. Data are presented as the mean ± standard error of the mean (SEM). *p < 0.05, **p < 0.01, ***p < 0.001, ****p < 0.0001.

To conclusively demonstrate the upstream regulatory role of AMPK in this signaling pathway, we performed rescue and inhibition experiments using the AMPK activator AICAR and the specific inhibitor Compound C, respectively (Fig 8). Under co-stimulation with LPS and PA, pharmacological activation of AMPK by AICAR significantly restored PPARα expression and effectively prevented the loss of Occludin and Claudin-1 expression. Conversely, in the absence of LPS/PA stress, direct inhibition of AMPK with Compound C recapitulated the detrimental effects induced by the combined treatment, leading to marked suppression of PPARα and downregulation of barrier proteins. Collectively, these in vitro data demonstrate that lipotoxicity and inflammation compromise intestinal barrier function by functionally disrupting the protective AMPK/PPARα metabolic signaling axis.

**Fig 8:**
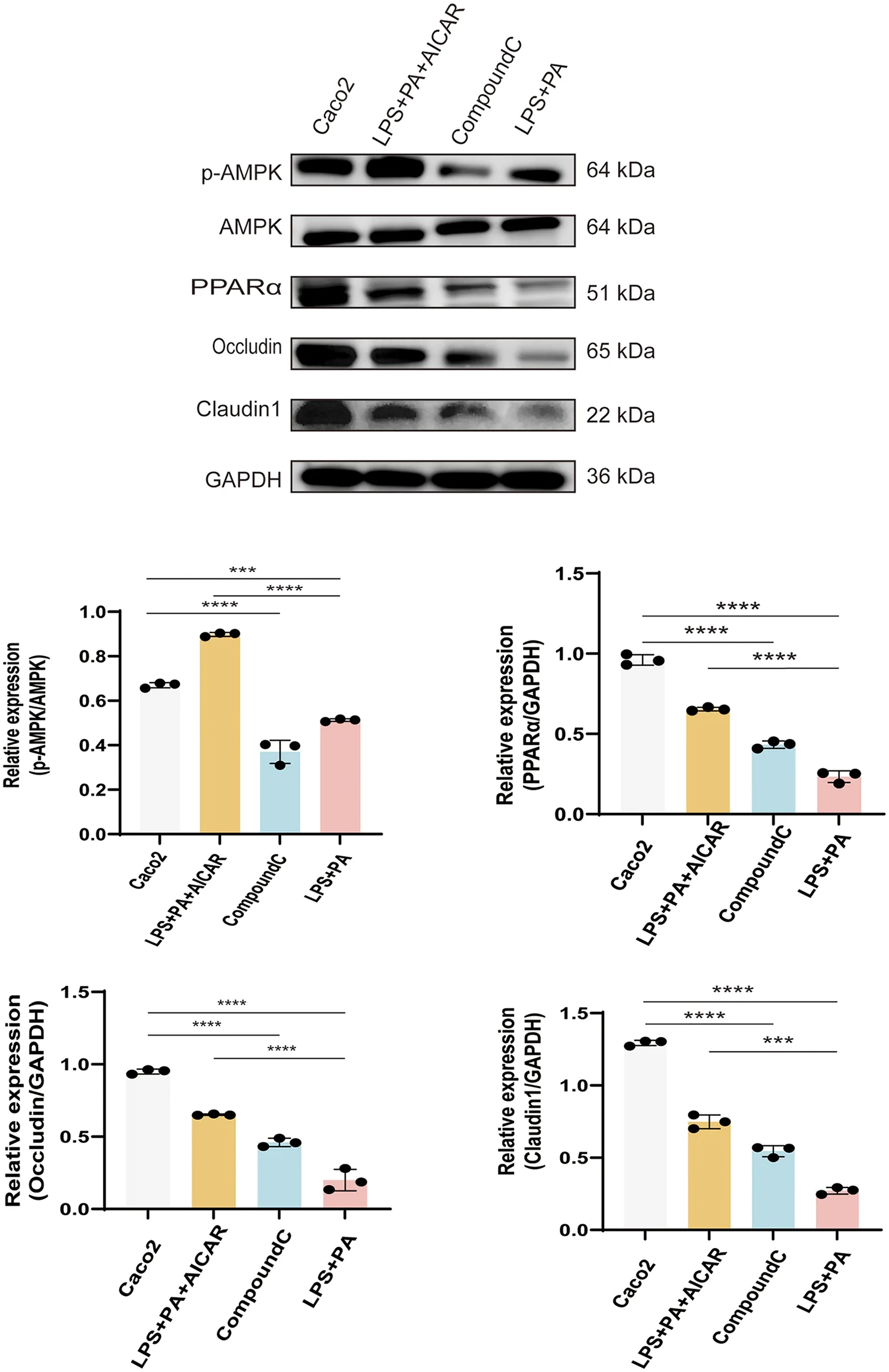
Representative Western blot images showing protein expression levels of p-AMPK, AMPK, PPARα, Occludin, and Claudin-1 in Caco-2 cells from each group. GAPDH was used as a loading control for normalization (n = 3). Quantitative analysis of protein expression levels in each group. Data are presented as the mean ± standard deviation (SD). ****p < 0.0001.

### Lipotoxicity and inflammatory stress synergistically induce ROS overproduction, which mediates the suppression of AMPK activation

Having determined that combined in vivo treatment with a high-fat diet and DSS leads to epithelial metabolic and barrier dysfunction, we next sought to elucidate the direct upstream molecular triggers in vitro. Given that lipotoxicity and inflammation are closely associated with oxidative stress, we assessed intracellular ROS levels in Caco2 cells using fluorescence microscopy (Fig 9A). Compared with the control group, treatment with either LPS or PA alone moderately elevated intracellular ROS levels, whereas combined LPS and PA exposure induced a massive burst of ROS production. This oxidative stress was almost completely neutralized by the antioxidant NAC. We then investigated whether this ROS accumulation accounted for the paradoxical inactivation of the AMPK pathway observed in our model. Western blot analysis revealed that cotreatment with LPS and PA significantly suppressed AMPK phosphorylation in Caco2 cells. Importantly, scavenging ROS with NAC in the presence of both LPS and PA restored AMPK phosphorylation levels (Fig 9B). Collectively, these data indicate that lipotoxicity and inflammation directly compromise host intestinal epithelial cells by triggering an oxidative storm, which in turn acts as an upstream mediator that aberrantly suppresses the AMPK metabolic axis.

**Fig 9:**
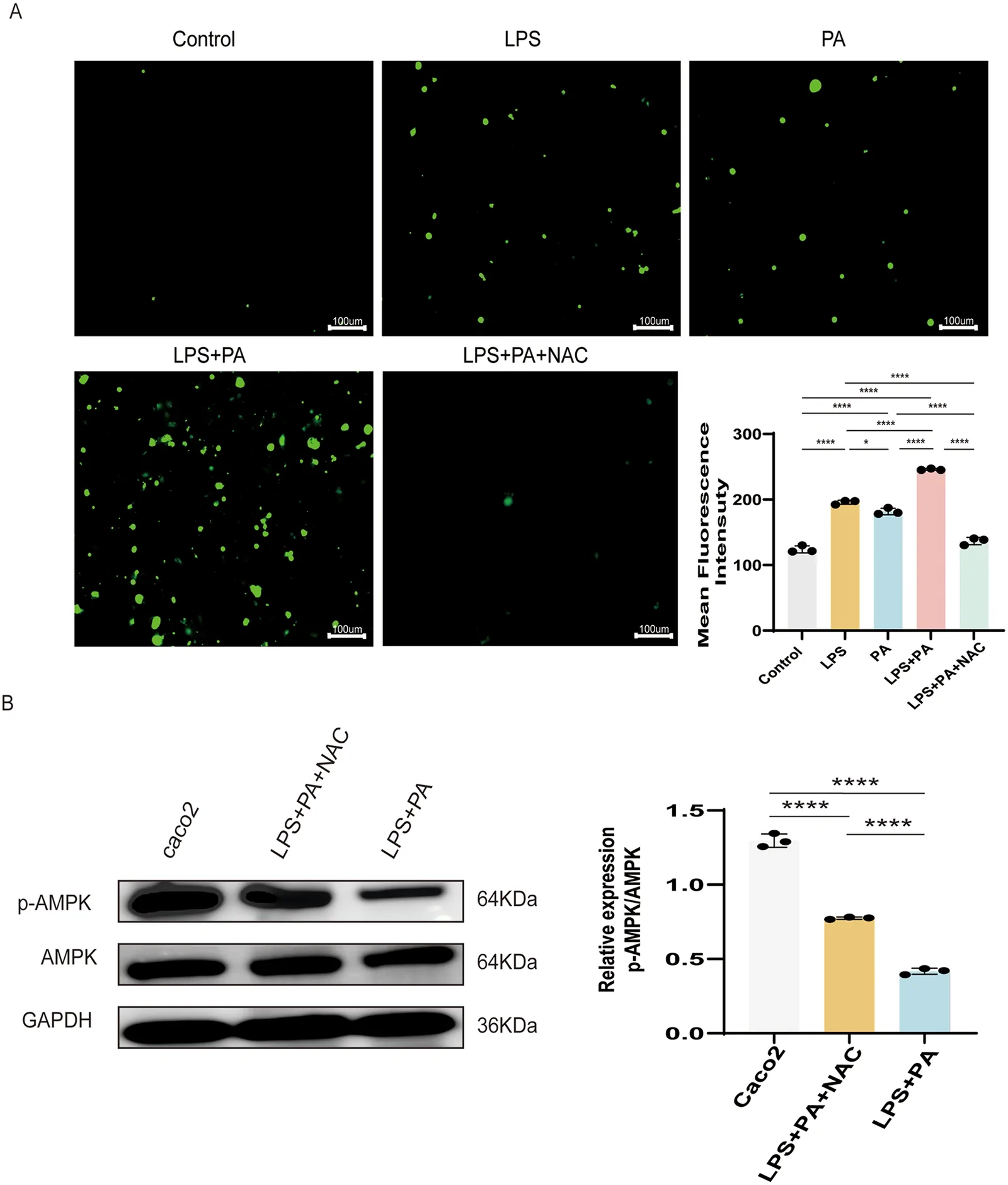
(A) Representative fluorescence microscopy images of ROS in each treatment group. Scale bar: 100 μm. Quantitative analysis of mean fluorescence intensity in each group, shown as a histogram. *p < 0.05, ****p < 0.0001. Data are representative of three independent experiments. (B) Representative Western blot images showing protein expression levels of p-AMPK and AMPK in Caco-2 cells from each group. GAPDH was used as a loading control for normalization (n = 3). Quantitative analysis of protein expression levels in each group. Data are presented as the mean ± standard deviation (SD). ****p < 0.0001.

### High-fat intervention significantly exacerbates the decrease in MMP in inflammation-induced Caco-2 cells

To evaluate the effects of high-fat and inflammatory induction on the mitochondrial function of Caco-2 cells, we utilized the JC-1 fluorescent probe to detect the levels of MMP across all groups. When MMP is high, JC-1 accumulates in the mitochondrial matrix to form aggregates, emitting red fluorescence; conversely, when the membrane potential decreases, JC-1 exists as monomers in the cytoplasm, emitting green fluorescence. Fluorescence microscopy revealed that control cells exhibited intense red fluorescence alongside minimal green fluorescence, indicating the maintenance of a normal, high mitochondrial membrane potential (MMP). In contrast, individual treatment with either LPS or PA led to a reduction in red fluorescence and a concomitant increase in green fluorescence, reflecting evident mitochondrial depolarization. Notably, in cells co-treated with LPS and PA, the red signal was nearly abolished, while the green signal peaked, demonstrating a severe collapse of the MMP. These microscopic observations were further validated by the quantitative analysis of the red-to-green fluorescence ratio across all groups (Fig 10). Taken together, both LPS and PA alone are sufficient to initiate mitochondrial depolarization, and their combined exposure drastically exacerbates this loss, resulting in pronounced mitochondrial dysfunction.

**Fig 10:**
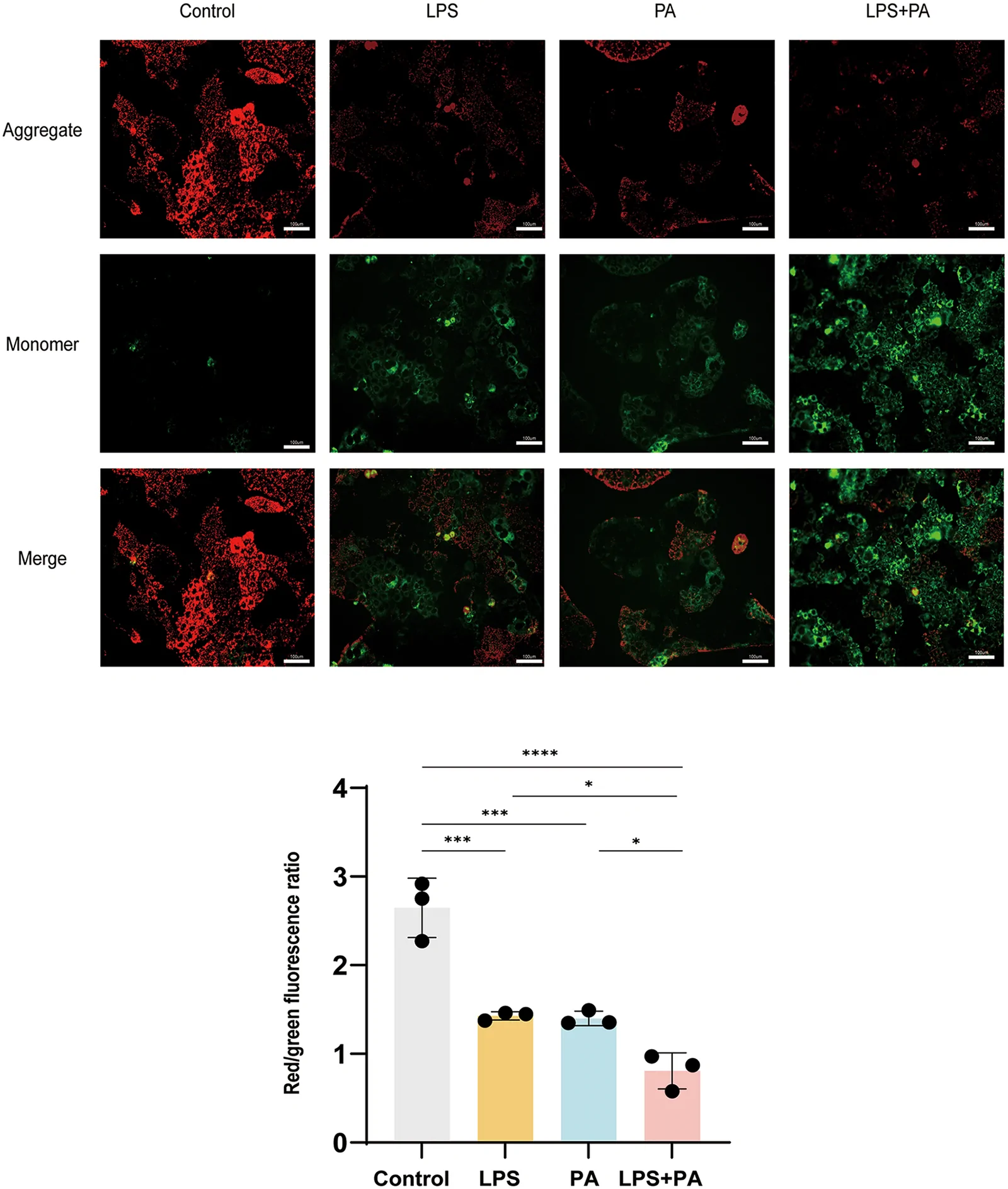
Representative fluorescence microscopy images of Caco-2 cells stained with the JC-1 fluorescent probe in each treatment group. Scale bar: 100 μm. Quantitative analysis of the red/green fluorescence intensity ratio in each group. *p < 0.05, ***p < 0.001, ****p < 0.0001. Data are representative of three independent experiments.

## Discussion

In this study, we utilized a DSS-induced mouse model in combination with an in vitro Caco-2 cell model to systematically investigate the specific pathological mechanisms by which a HFD exacerbates the progression of UC. We found that HFD significantly aggravated clinical and histological damage to the colonic mucosa. Mechanistically, this exacerbation is attributed to the synergistic effect of lipid overload-induced lipotoxicity and inflammatory signals derived from gut microbiota dysbiosis. These two factors collectively inflict severe damage on intestinal epithelial cells, ultimately disrupting the mucosal barrier by inducing mitochondrial oxidative stress.

HFD exposure severely worsened DSS-induced colitis, as reflected by accelerated weight loss, higher DAI scores, and extensive crypt destruction. Notably, even HFD intake alone induced mild colon shortening in healthy mice without DSS treatment. This finding is consistent with the concept of metabolic intestinal disease. This concept describes a condition in which, in the absence of typical immune activation or organic ulceration, overnutrition (especially lipid overload) triggers intestinal epithelial energy imbalance, mitochondrial dysfunction, and microstructural remodeling [25]. Similar subclinical bowel impairments are frequently observed in obesity, type 2 diabetes, and NAFLD [26,27]. Our findings thus reinforce how baseline dietary habits prime the gut for IBD susceptibility [28,29].

At the molecular level, aberrant lipid metabolism acts as the primary pathological driver. HFD feeding elevated systemic lipid profiles (TC, TG, and LDL-C), while in vitro palmitic acid (PA) exposure resulted in massive lipid droplet accumulation in Caco-2 cells. This lipotoxicity resembles the well-documented mechanisms in NAFLD pathogenesis [24]. Our findings suggest that lipid accumulation in intestinal epithelial cells may represent an early event in UC progression.

Meanwhile, long-term HFD feeding profoundly altered the gut microbiota. 16S rRNA gene sequencing revealed that HFD significantly reduced bacterial α-diversity and promoted the overgrowth of potentially pathogenic taxa, such as the phylum Proteobacteria. Notably, while microbiome-based functional predictions suggested alterations in multiple metabolic pathways, these predictions primarily reflect the intrinsic metabolic potential of the bacterial community itself and cannot be directly equated with changes in host eukaryotic signaling pathways. Therefore, our data indicate that gut dysbiosis does not act as a direct causal pathway regulating specific host gene expression; rather, we propose that the disturbed microbial microenvironment, functioning as a persistent pro-inflammatory signal, synergizes with HFD-induced intracellular lipotoxicity. Under DSS challenge, pro-inflammatory stimuli derived from the dysbiotic microbiota and the host’s innate lipotoxicity collectively exacerbate the metabolic stress on intestinal epithelial cells, ultimately leading to the aggravation of ulcerative colitis.

Prior bioinformatic analyses of human UC patients have robustly identified the inhibition of the PPAR pathway and down-regulation of its downstream fatty acid-metabolizing enzymes as a hub in mucosal metabolic disorders. In striking alignment with these clinical observations, our in vivo models demonstrated that HFD and the aforementioned dual insults strongly suppress PPARα expression. To elucidate the upstream drivers of this suppressed metabolic axis. In this study, we investigated the paradoxical inactivation of AMPK in intestinal epithelial cells when facing the aforementioned dual insults. Classical theory holds that decreased ATP levels, reflecting cellular energy deficiency, and mild ROS accumulation typically activate AMPK to maintain energy homeostasis [30]. However, in both our in vivo and in vitro studies, we observed ATP depletion accompanied by a marked decrease in p-AMPK levels [31,32]. This is because, although physiological ROS can activate AMPK [33], the oxidative stress burst triggered by severe lipotoxicity and inflammation impairs the activity of upstream kinases of AMPK or overactivates phosphatases, leading to its dephosphorylation [34,35]. In our in vitro experiments, we successfully rescued the suppressed p-AMPK levels by adding the ROS scavenger NAC, thereby providing causal evidence for this aberrant ROS-induced paradoxical inactivation of AMPK.

Lipid metabolism disturbance not only triggers intracellular lipotoxicity but also directly damages mitochondrial structure and function [36,37]. Using TEM, we observed severe ultrastructural changes in intestinal epithelial mitochondria from the HD group. This damage led to an energy crisis and redox imbalance, as seen in marked ATP depletion and excessive ROS production [38]. NDUFS2 (NADH dehydrogenase [ubiquinone] iron-sulfur protein 2) is a nuclear-encoded core subunit of mitochondrial respiratory chain complex I (MRC I) [39]; it regulates cell growth, membrane integrity, ROS generation, and ATP synthesis [40]. Meanwhile, MTCO2, a key factor controlling oxidative phosphorylation and aerobic energy production coupled with ATP synthesis [41,42], works alongside NDUFS2 to maintain normal ATP production [43]. When these subunits become dysfunctional, the mitochondria depolarize. Such depolarization then worsens FAO dysfunction, cutting off the energy supply needed for intestinal epithelial cell proliferation and repair. The epithelium thus loses the energy required for mucosal repair, while the accompanying ROS surge directly disrupts tight junctions [44]. In summary, intestinal inflammation under a high-fat diet damages mitochondrial structure, suppresses the NDUFS2/MTCO2-centered electron transport chain and oxidative phosphorylation, triggers an energy crisis and ROS burst, and ultimately compromises the integrity of the intestinal epithelial barrier.

The mechanistic insights from this study hold important translational potential. First, at the epidemiological level, patients with metabolic syndrome have a markedly higher risk of developing UC [45]. This increased risk may be linked to HFD-induced gut metabolic disturbances and mitochondrial dysfunction. Targeting the AMPK-PPARα axis emerges as a promising adjunctive therapy. In this study, we employed an in vitro cell model to verify the reversal effect of AMPK activation on pathological phenotypes. Treatment with the specific AMPK agonist AICAR effectively reversed the LPS and PA induced suppression of p-AMPK, accompanied by restored expression of PPARα and intestinal barrier proteins. These findings provide evidence that AMPK inactivation serves as a driving event in HFD-exacerbated UC, and strongly support therapeutic strategies targeting this axis. Furthermore, it has been reported that AMPK activators such as metformin can ameliorate experimental colitis by enhancing barrier function and modulating macrophage polarization [46]. Fenofibrate, a PPARα agonist, alleviates UC symptoms and reduces inflammation in both preclinical models and clinical studies [23,47]. AMPK activators such as metformin improve experimental colitis by enhancing barrier function and modulating macrophage polarization [47]. In our study, HFD suppressed this axis, so it is reasonable to propose that pharmacological reactivation of AMPK or PPARα might ameliorate HFD-exacerbated UC. Furthermore, our findings highlight mitochondrial homeostasis as a key therapeutic target in HFD-aggravated UC. To reduce mitochondrial damage, organelle-specific interventions should be prioritized; mitochondria-targeted antioxidants such as MitoQ could serve as a metabolic reprogramming strategy for UC patients with high-fat dietary habits [48]. Looking ahead, the treatment paradigm for IBD should evolve from merely suppressing inflammation or broad immunosuppression toward a more integrated approach that includes microbiome modulation, precision nutrition, restoration of epithelial bioenergetic metabolism, and broader cellular functional recovery.

### Limitations

First, the animal model employed was DSS-induced acute UC, which does not fully recapitulate the chronic relapsing–remitting characteristics of human UC. Second, in our analysis of the association between gut microbiota and exacerbated colitis, we did not include fecal microbiota transplantation (FMT) experiments in mice; therefore, caution should be exercised when drawing definitive causal inferences regarding the role of the microbiota in aggravating the disease phenotype. Third, the PICRUSt2 were inferred solely from 16S rRNA sequencing data and thus require further validation through metagenomics or metatranscriptomics. Finally, the in vitro experiments were conducted solely using Caco-2 cell monolayers, without incorporating immune cell co-culture to simulate the mucosal immune microenvironment. Future studies should integrate single-cell sequencing, organoid models, and clinical cohorts to validate the proposed mechanisms and to explore the specific roles of novel targets such as PPARα and AMPK in intestinal epithelial lipid metabolism.

## Conclusion

In summary, this study elucidates the molecular pathological mechanisms by which a HFD exacerbates UC. HFD-induced lipotoxicity, together with gut microbiota dysbiosis, suppresses the AMPK/PPARα metabolic axis in host intestinal epithelial cells. This suppression subsequently blocks fatty acid oxidation, promotes intracellular lipid accumulation and mitochondrial structural damage, and culminates in an ATP energy crisis that compromises the mucosal barrier. Importantly, using AMPK agonists, we demonstrated that reactivation of AMPK effectively reverses the aforementioned pathological processes. This study moves the management of IBD beyond traditional immunosuppression toward a comprehensive strategy that targets metabolism and restores cellular function. This provides a solid theoretical basis for developing new adjunctive therapies based on metabolic reprogramming, especially for UC patients with metabolic disturbances or those who habitually consume a HFD.

## Supporting information

S1 Raw Images X. (PDF)
