# Supplementary material for: Impaired fatty acid oxidation and mitochondrial injury mediated by the AMPK/PPARα pathway: A key mechanism in high-fat diet-aggravated ulcerative colitis
Source: PLoS One. 2026 Oct 7;21(10):e0358354. doi: 10.1371/journal.pone.0358354 (PMC13645057; doi:10.1371/journal.pone.0358354)
Supplement: Supplementary file 1 [file pone.0358354.s001.pdf]

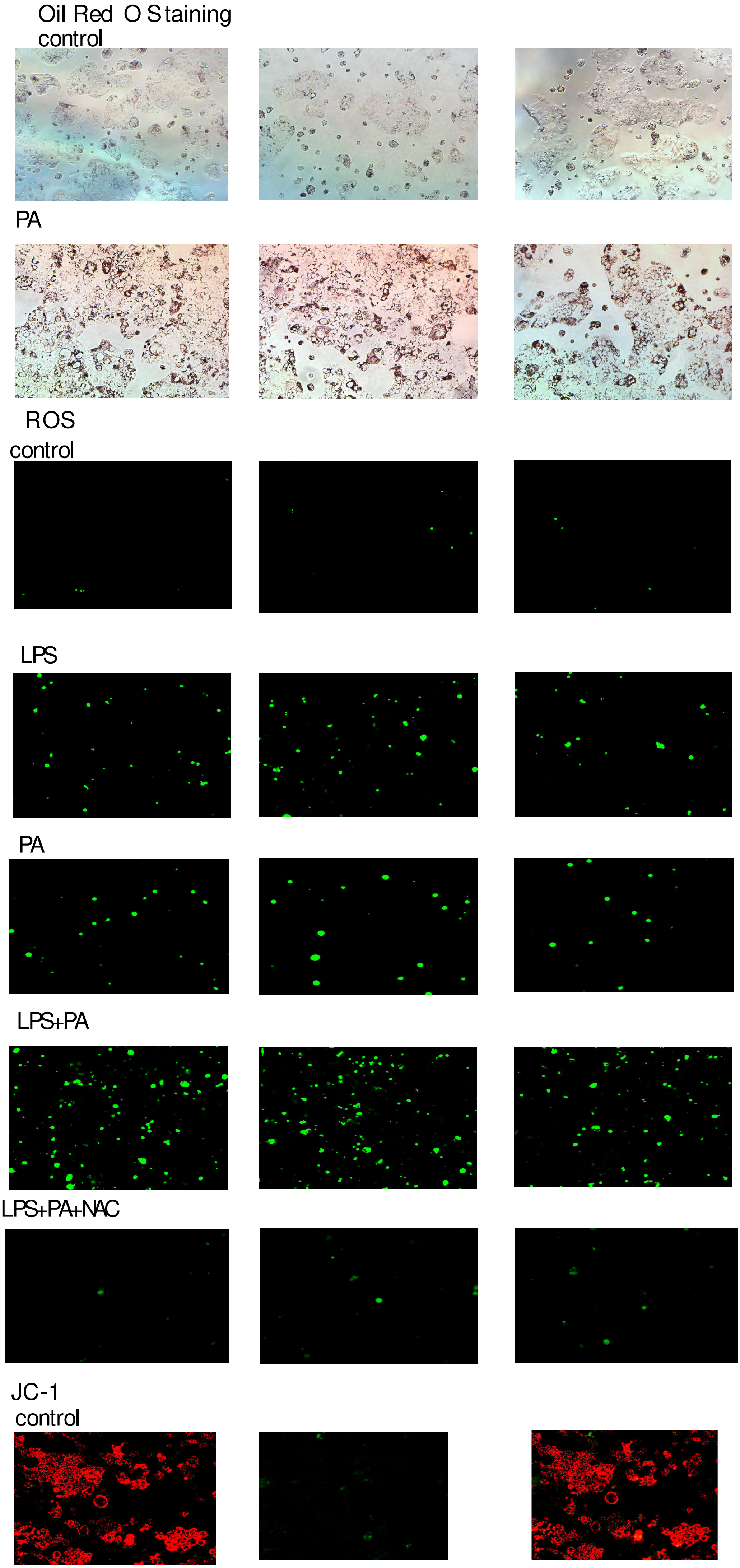
Oil Red O Staining
control
PA
ROS
control
LPS
PA
LPS+PA
LPS+PA+NAC
JC-1
control

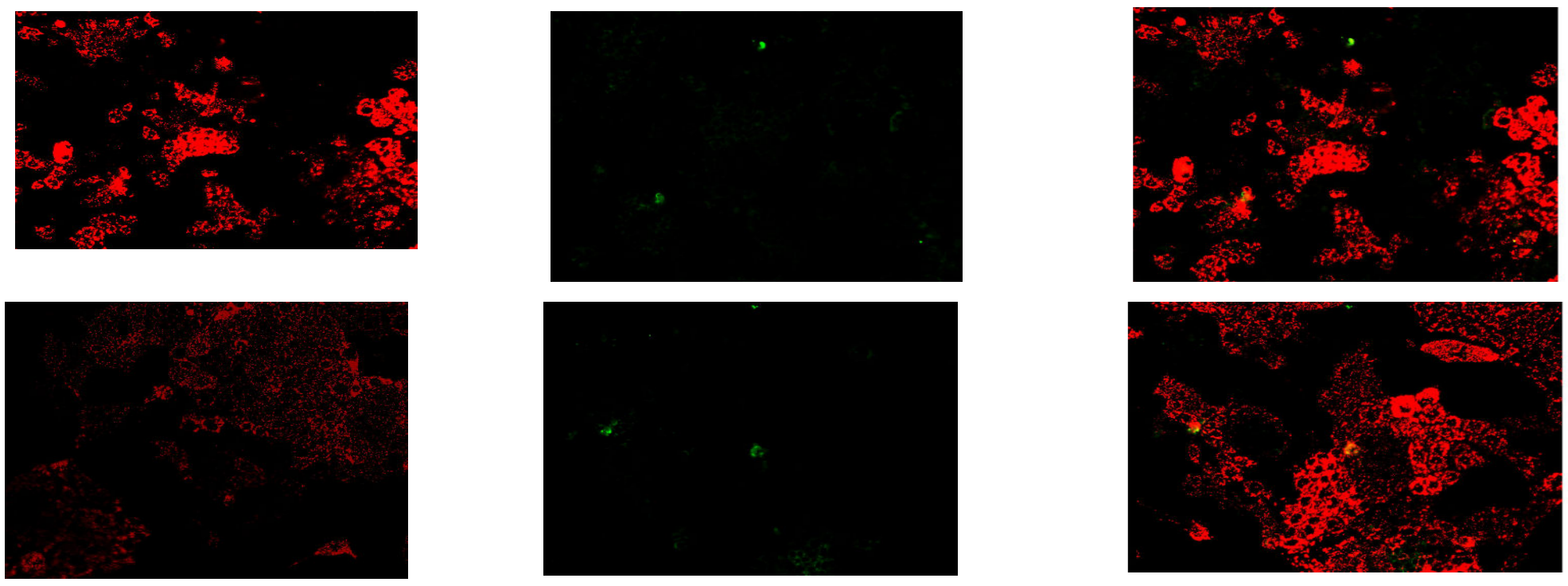

LPS

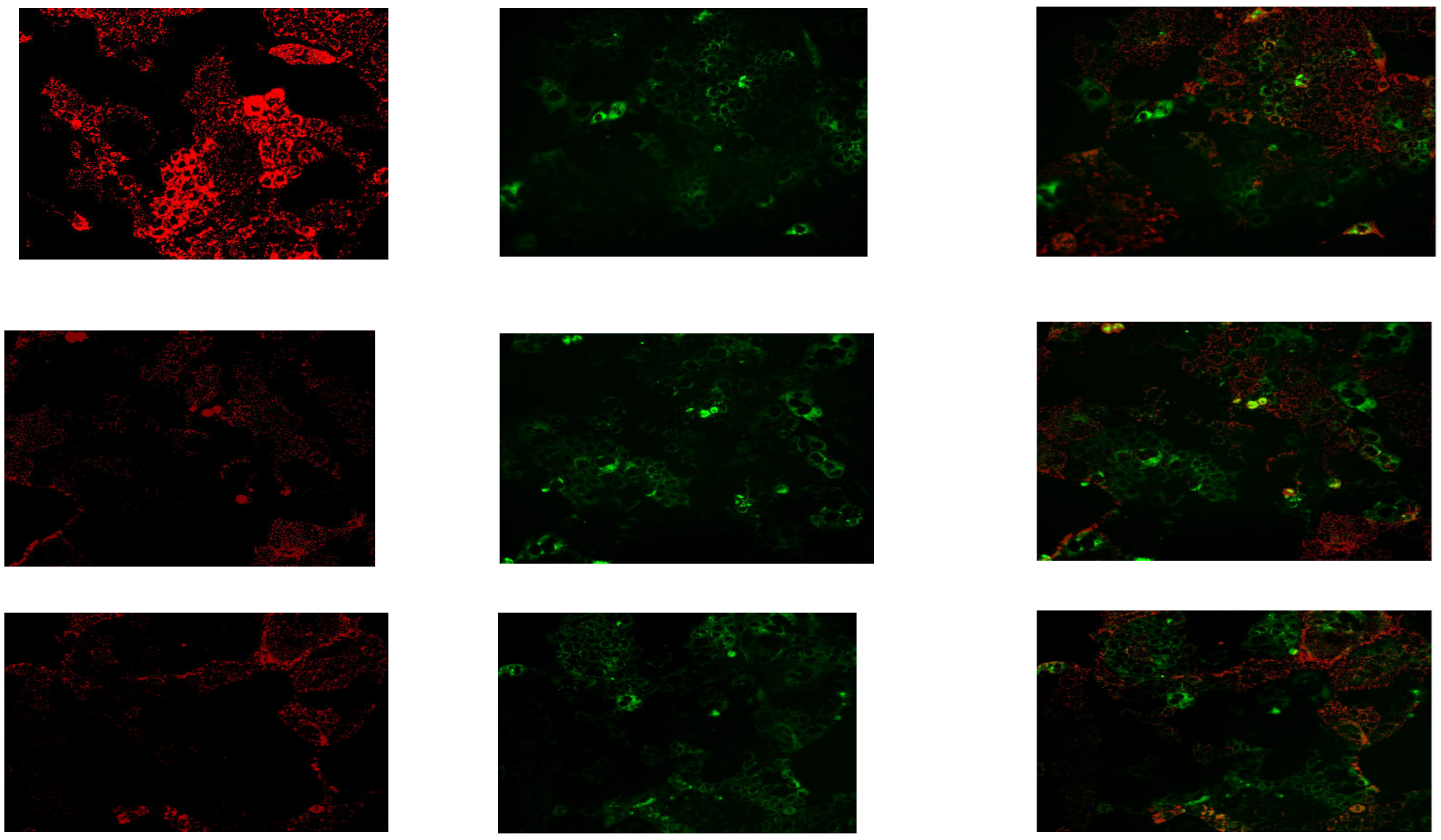

PA

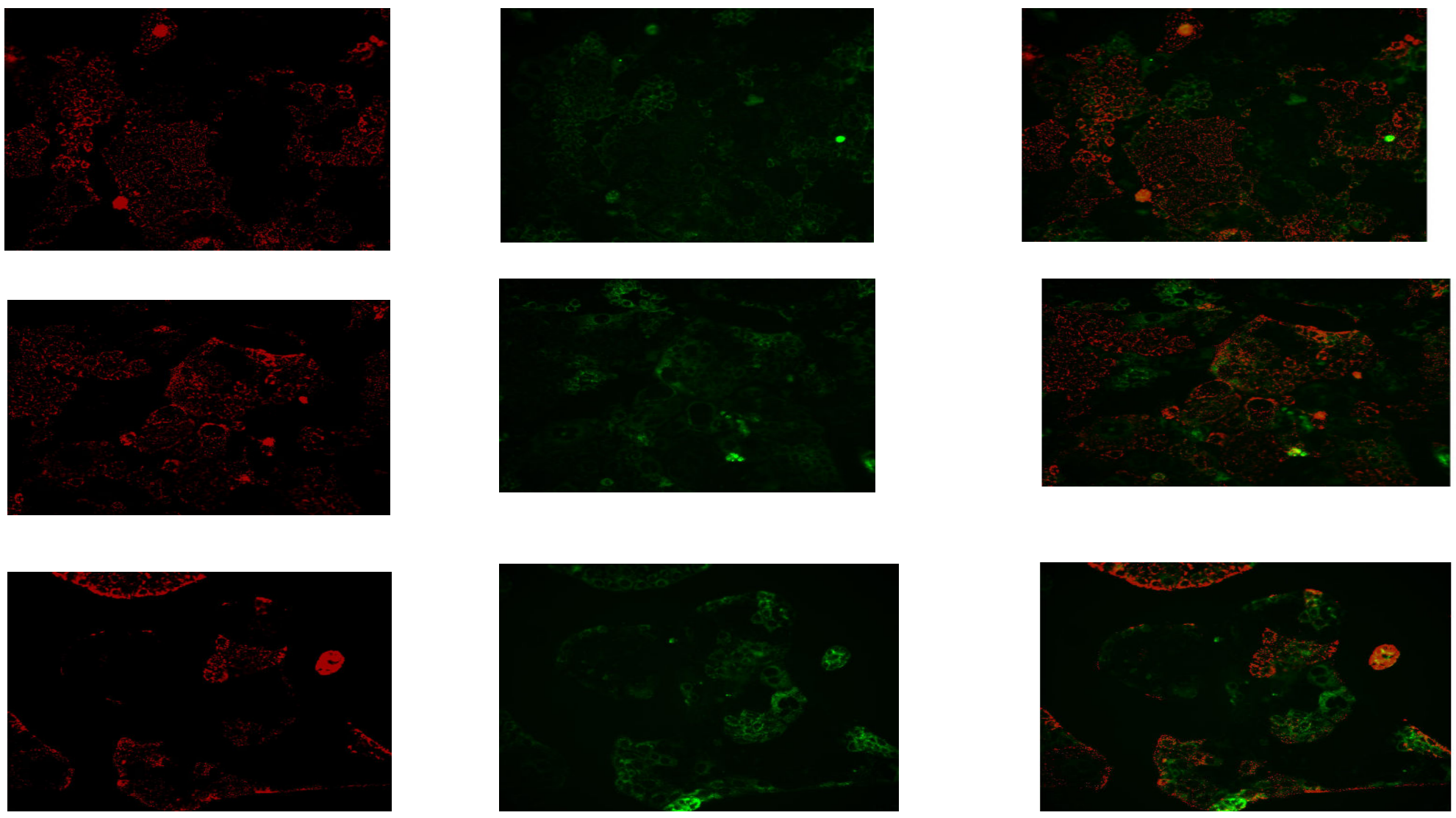

LPS+PA

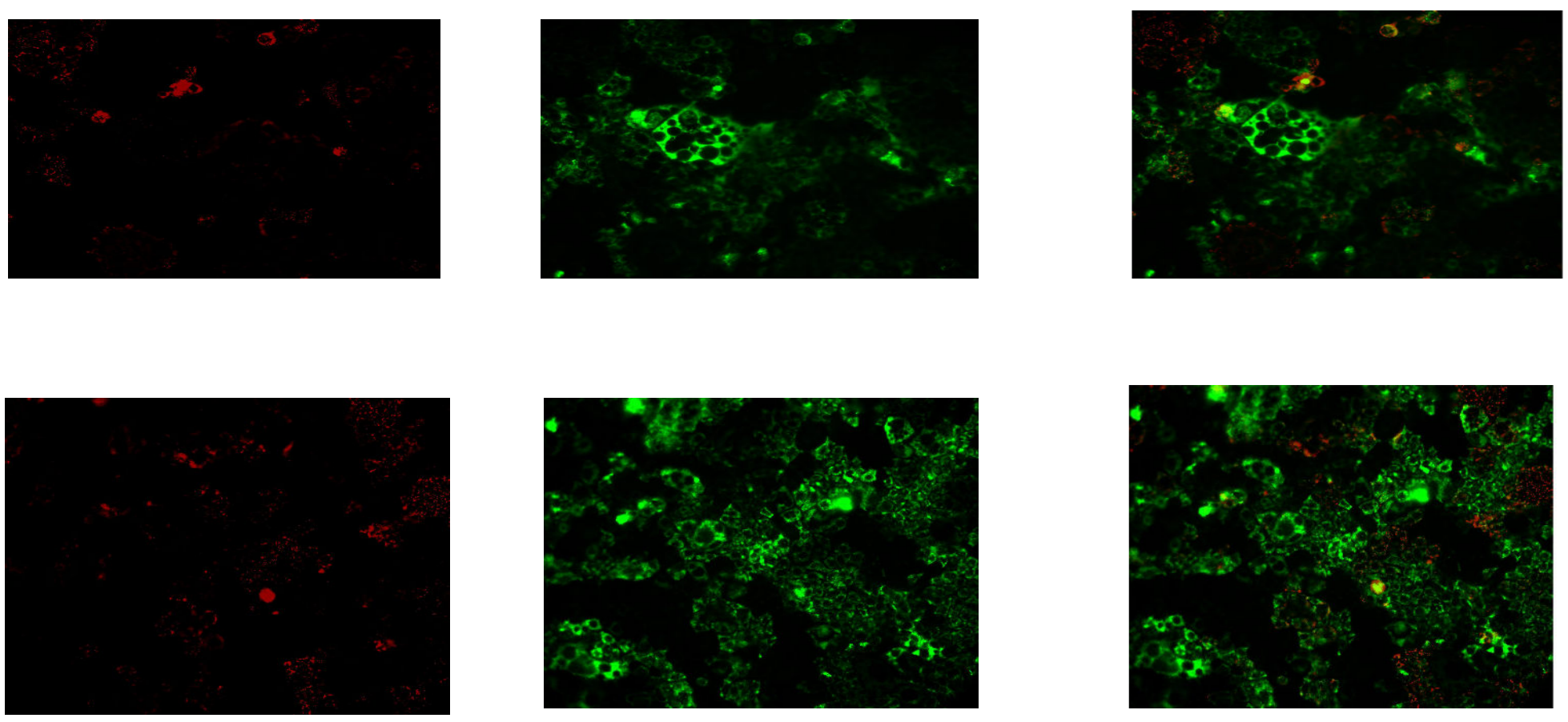

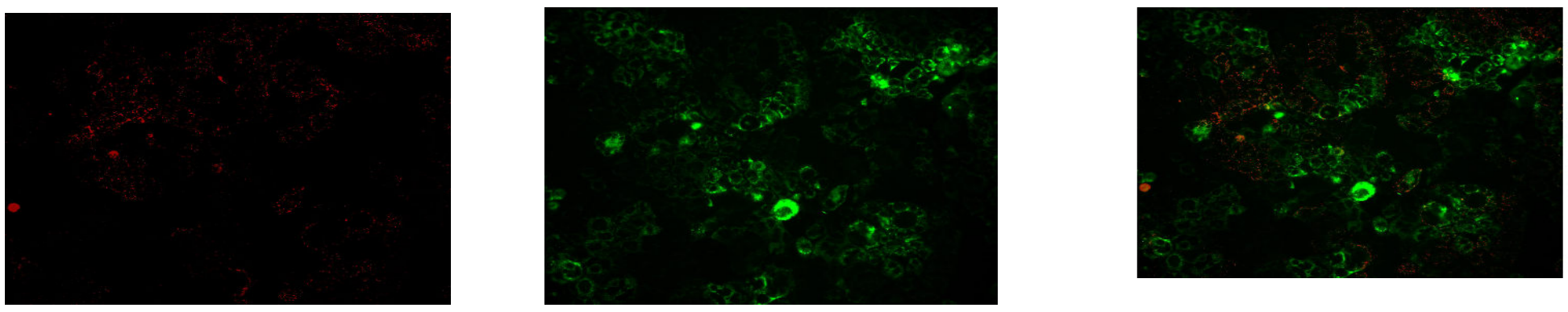

p-AMPK

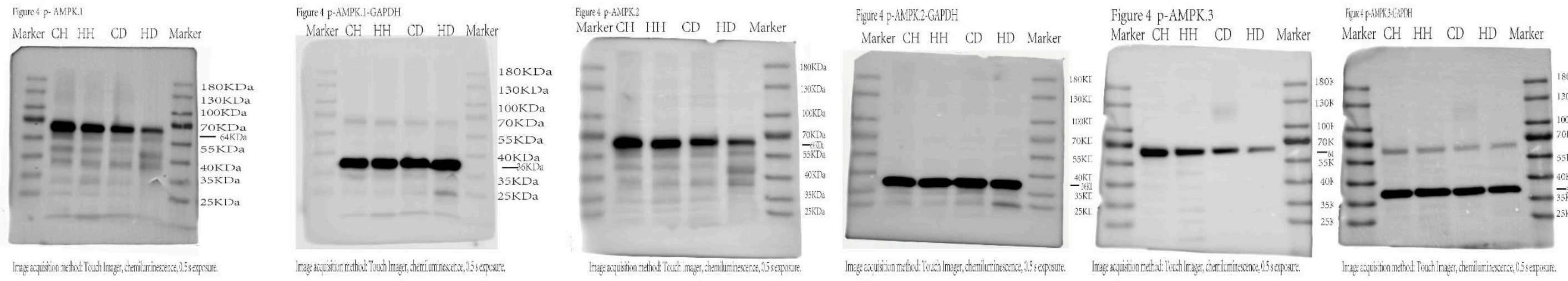

AMPK

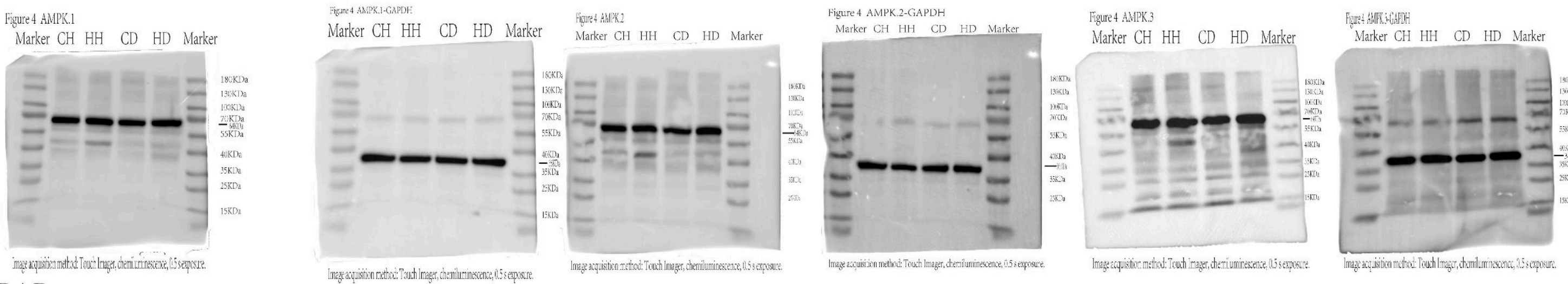

PPAR α

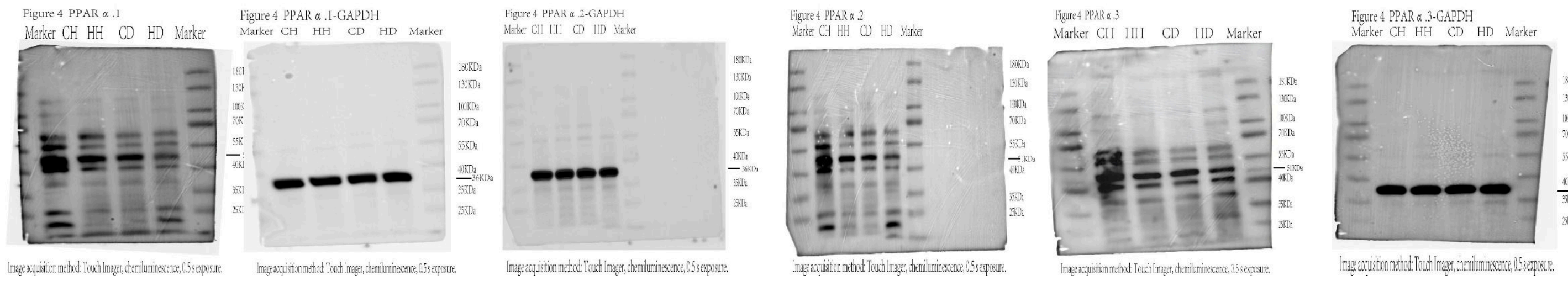

CPT1

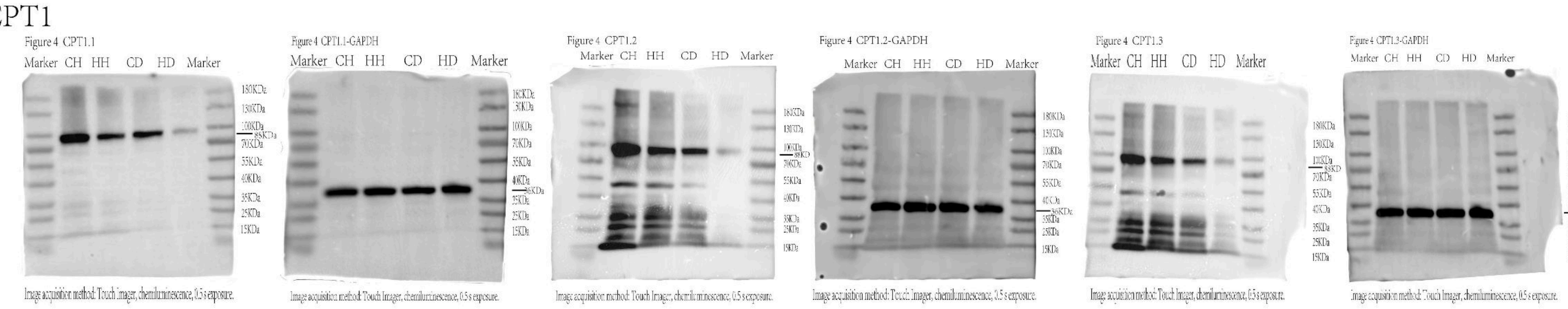

ACOX1

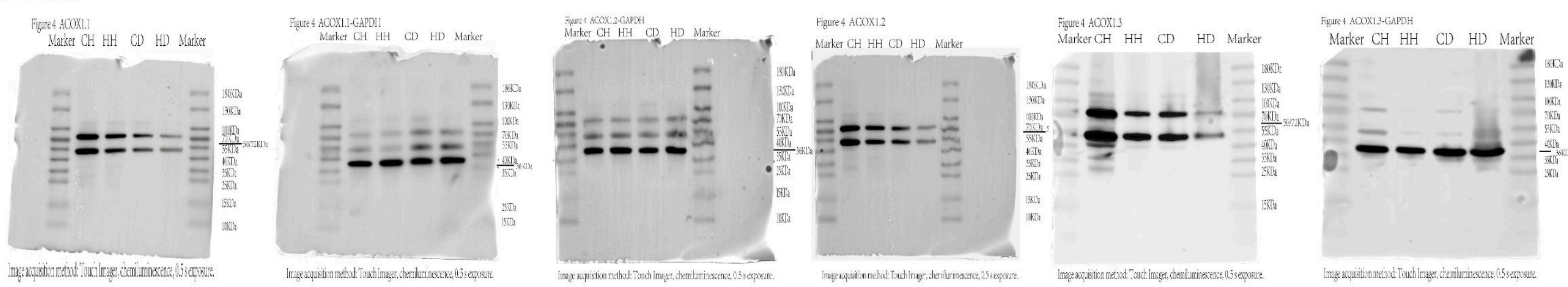

Occludin

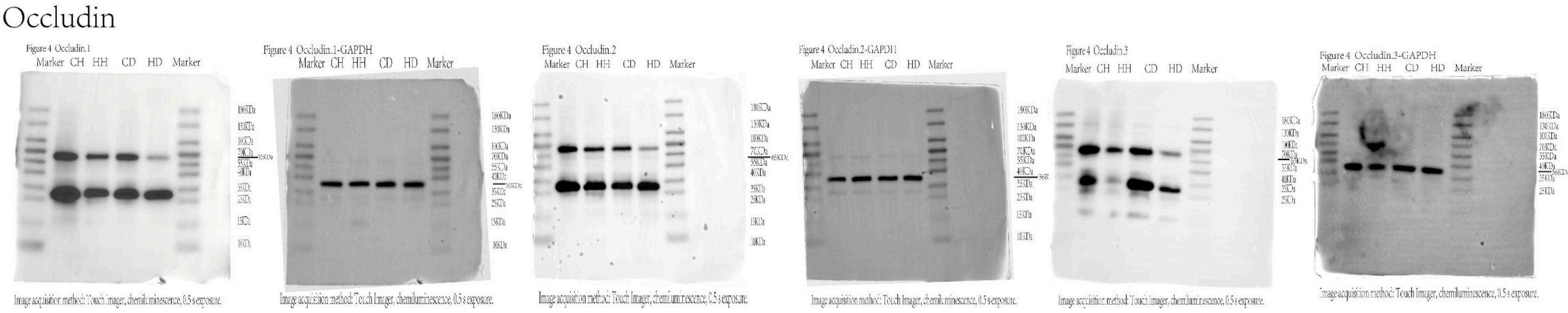

Claudin1

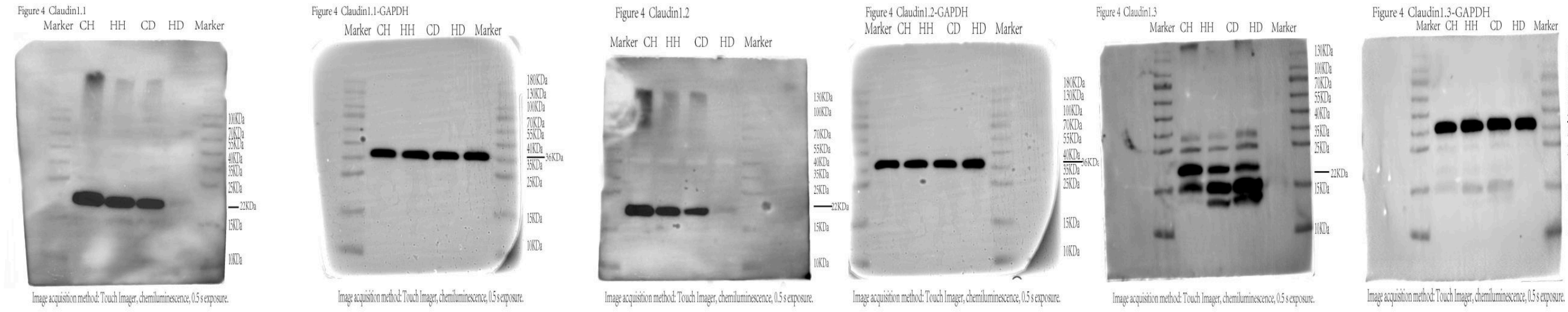

MTCO2

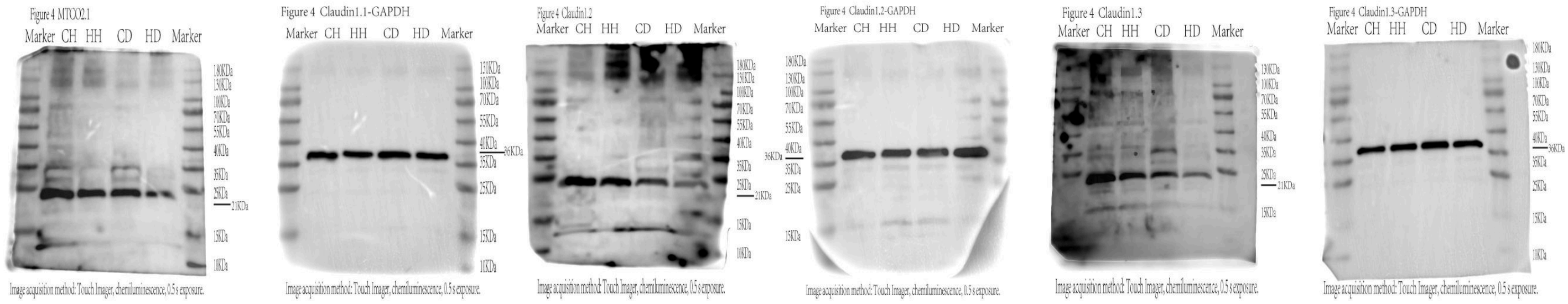

NDUFS2

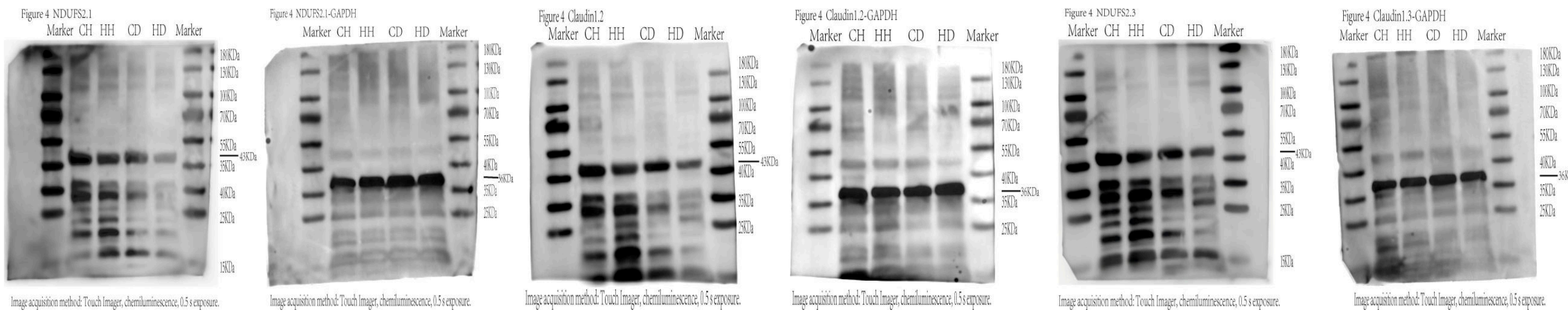

p-AMPK

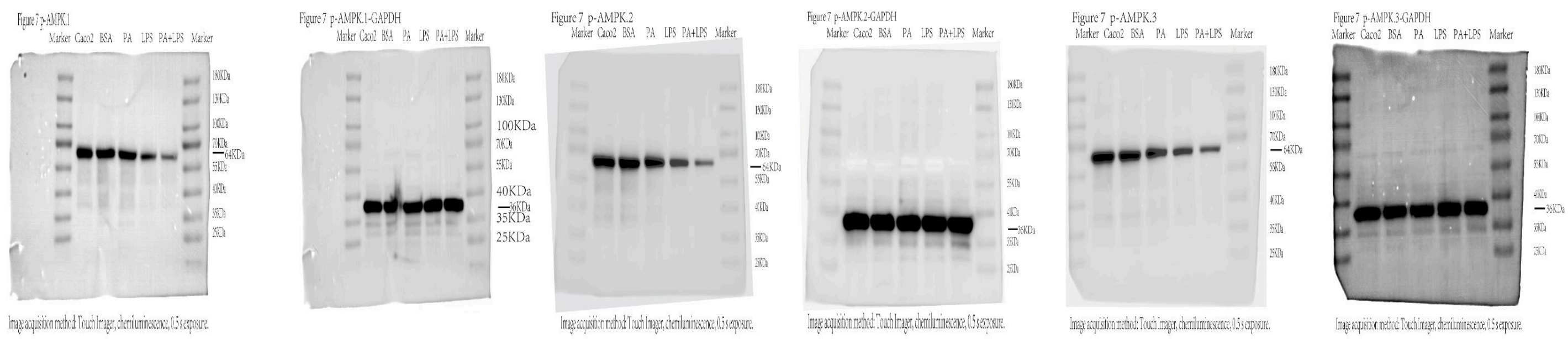

AMPK

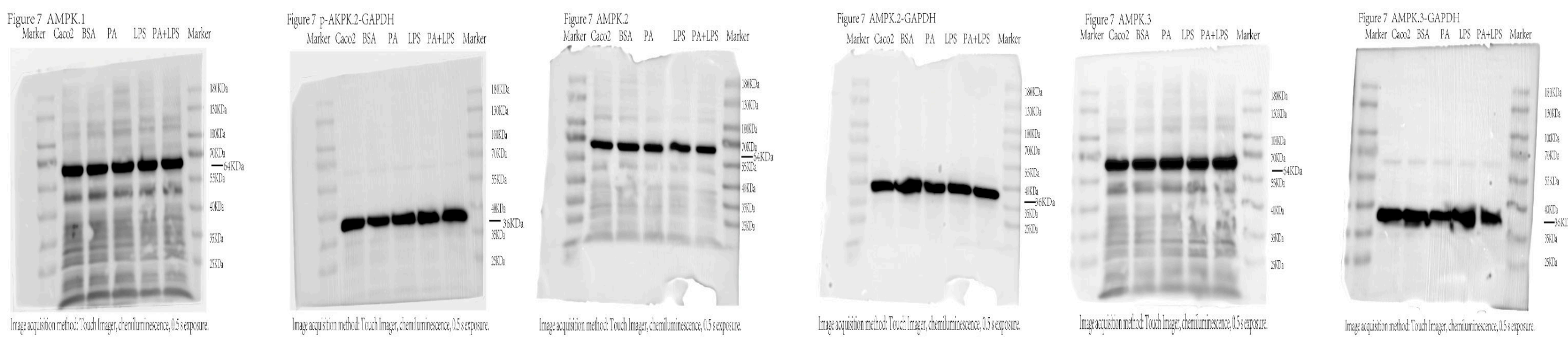

PPAR α

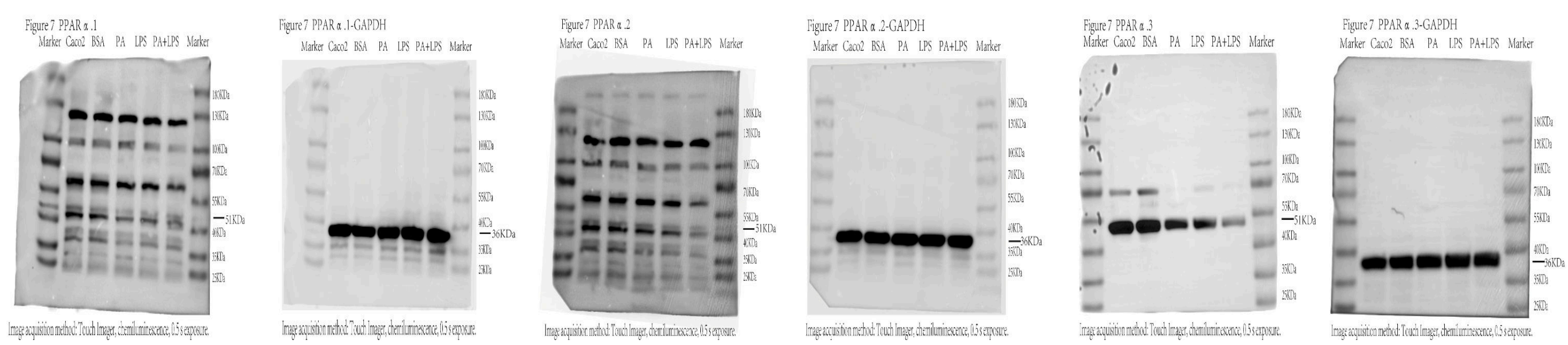

ACOX1

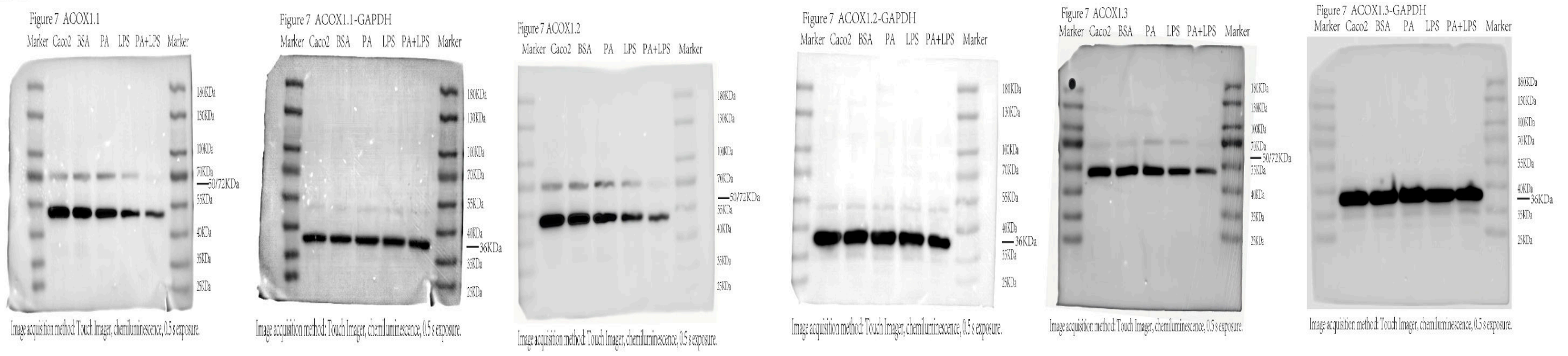

CPT1

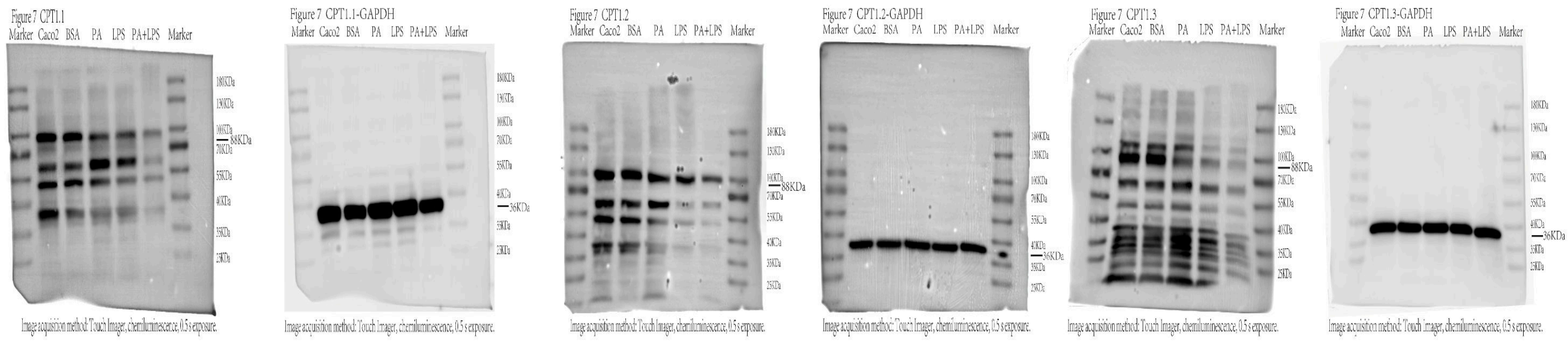

Occludin

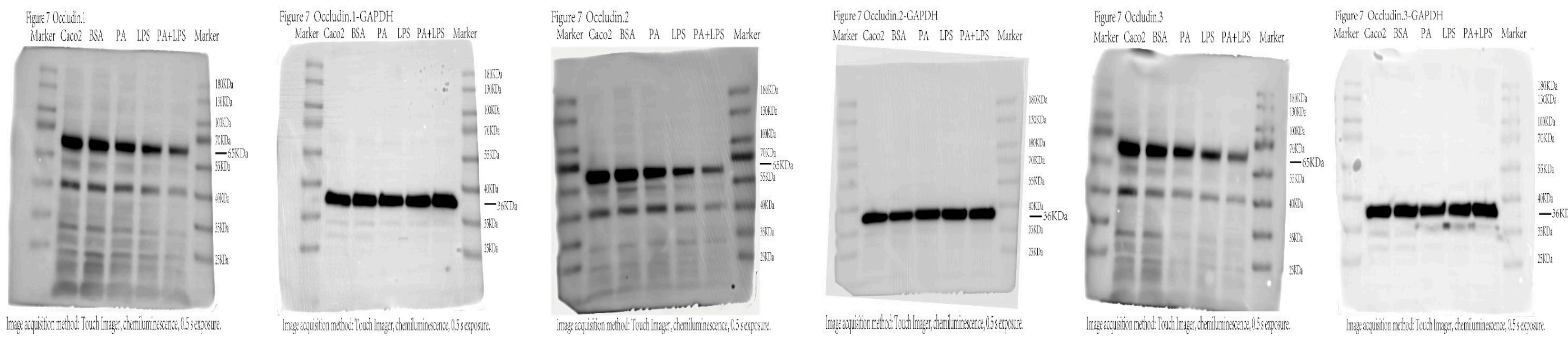

Claudin1

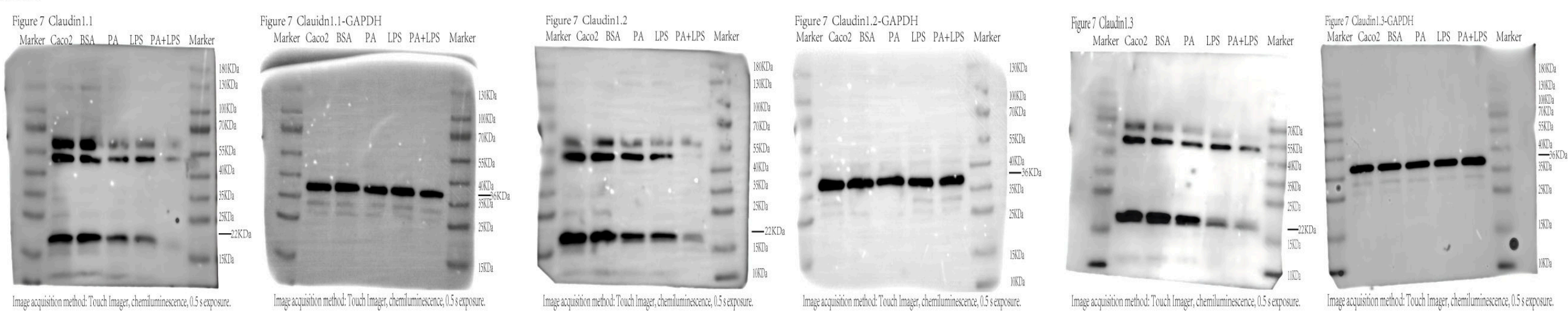

MTCO2

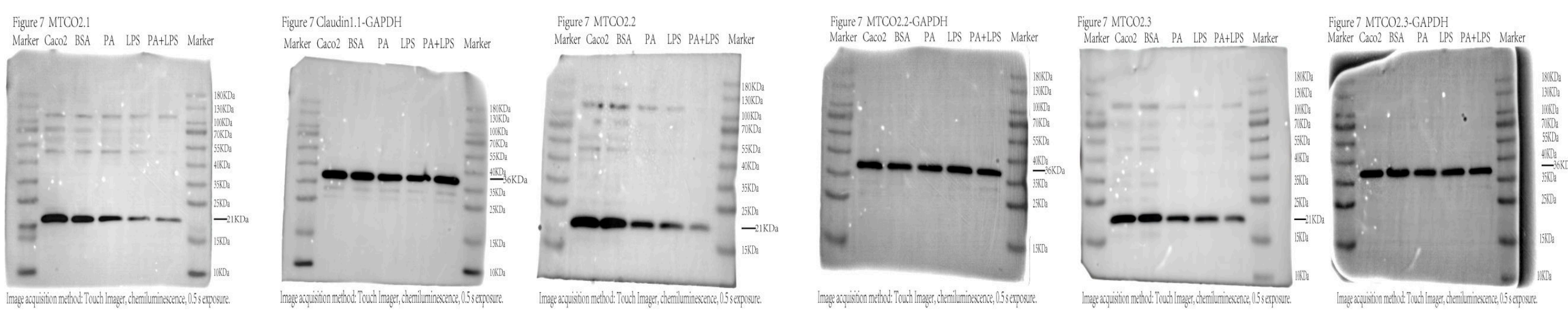

NDUFS2

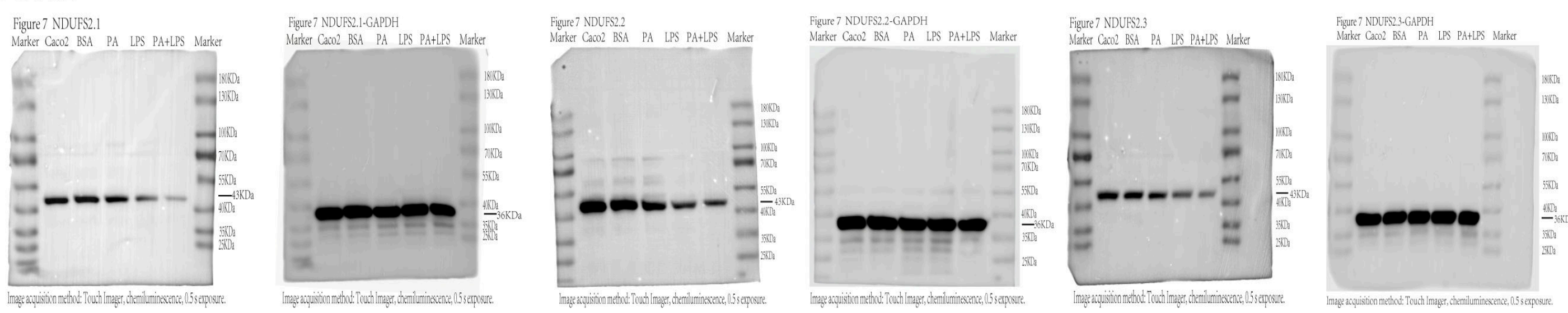

AMPK

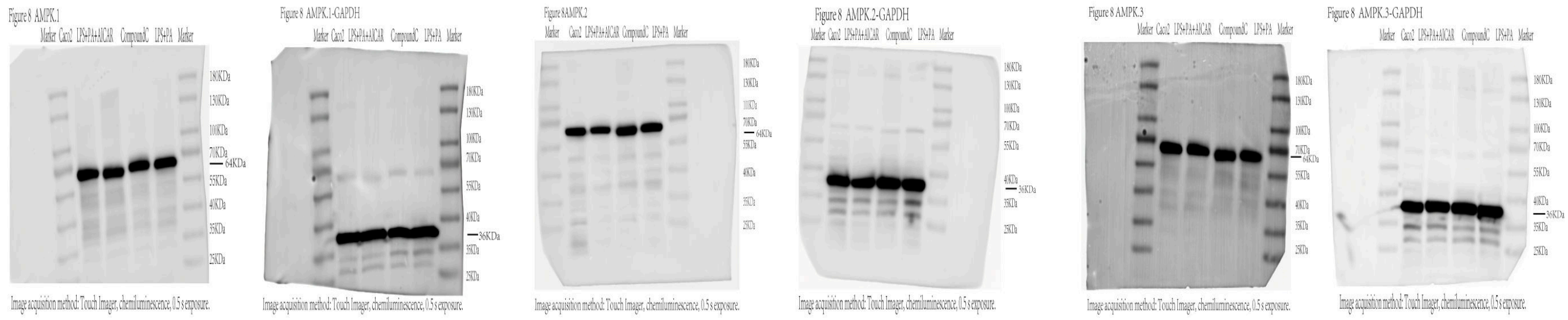

PPAR α

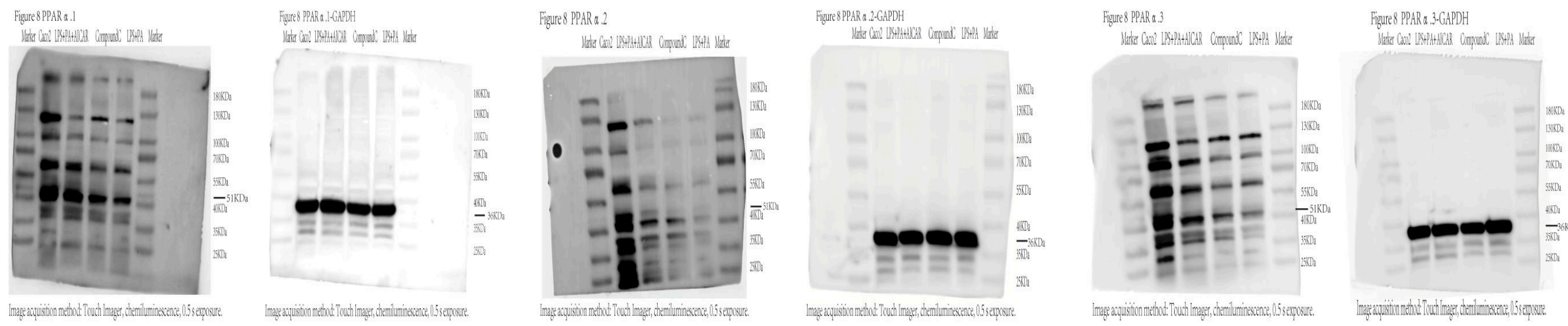

Occludin

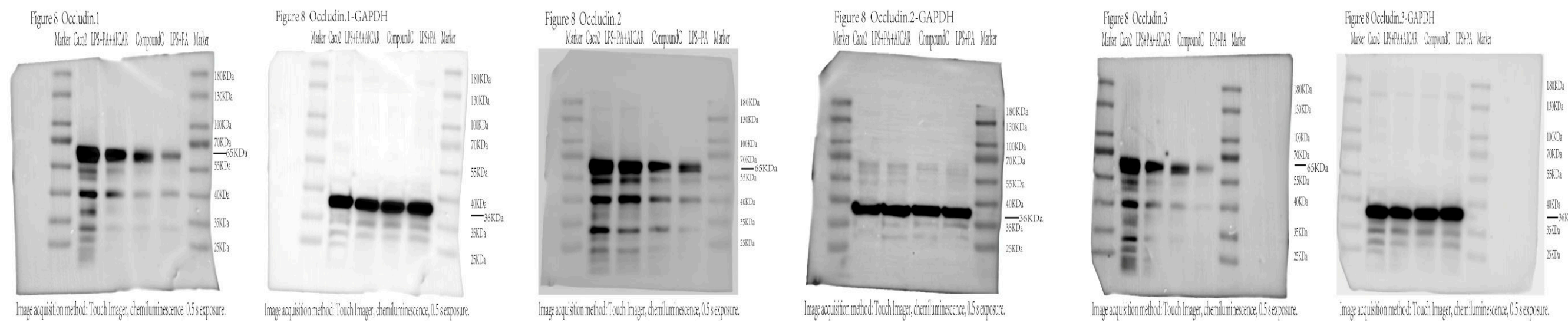

Claudin1

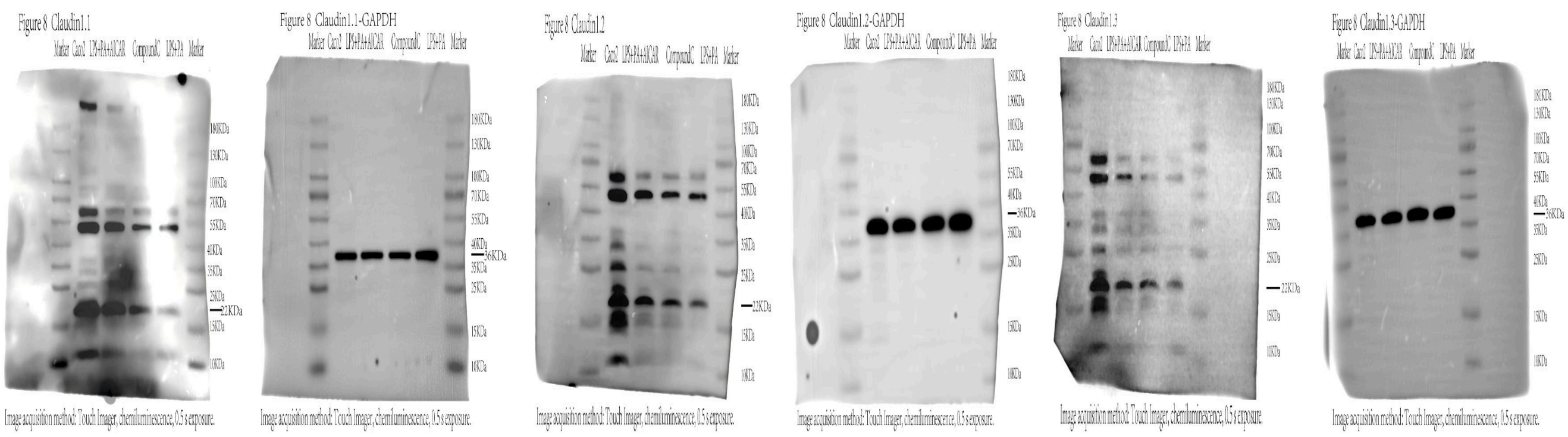

p-AMPK

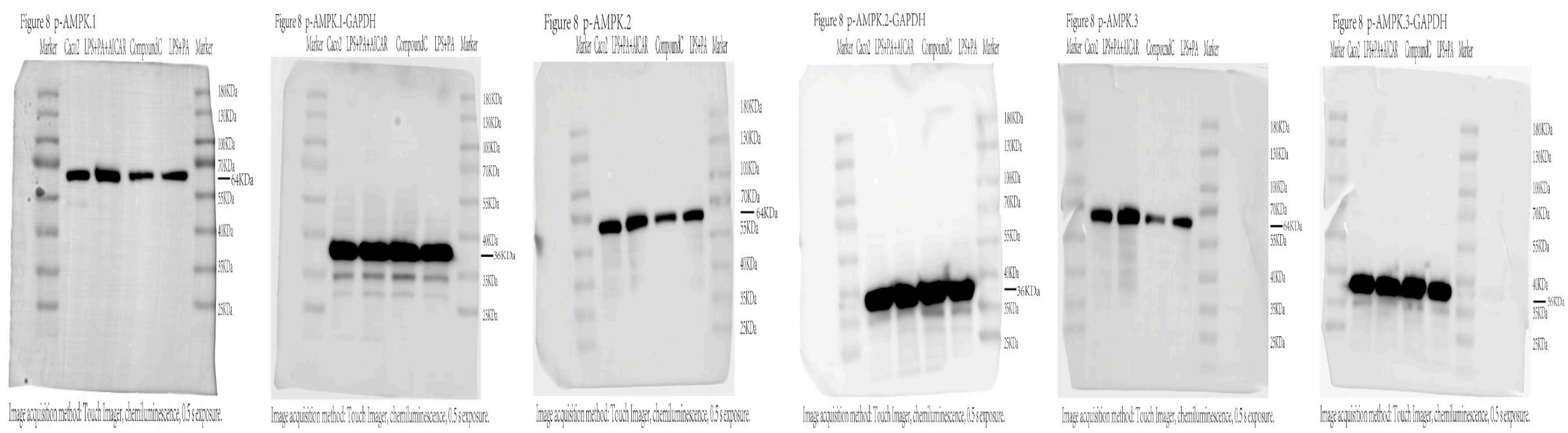

p-AMPK

Figure 9B p-AMPK.1
Marker Caco2 LPS+PA+NAC LPS+PA Marker

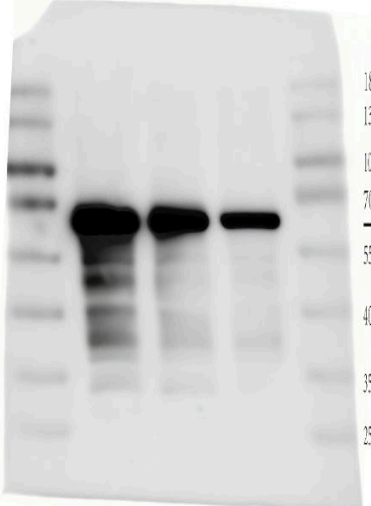

Image acquisition method: Touch Imager, chemiluminescence, 0.5 s exposure.

Figure 9B p-AMPK.1-GAPDH
Marker Caco2 LPS+PA+NAC LPS+PA Marker

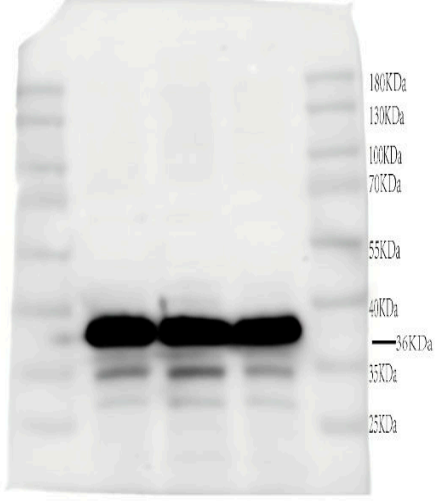

Image acquisition method: Touch Imager, chemiluminescence, 0.5 s exposure.

Figure 9B p-AMPK.2
Marker Caco2 LPS+PA+NAC LPS+PA Marker

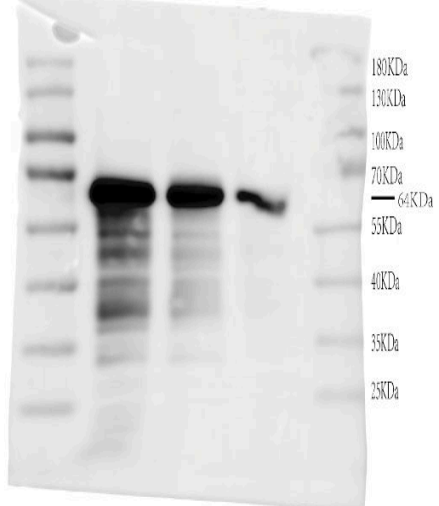

Image acquisition method: Touch Imager, chemiluminescence, 0.5 s exposure.

Figure 9B p-AMPK.2-GAPDH
Marker Caco2 LPS+PA+NAC LPS+PA Marker

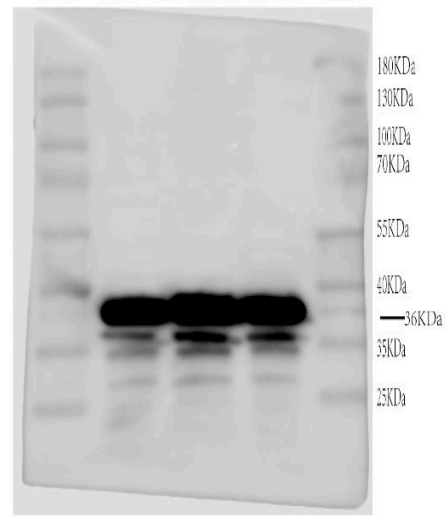

Image acquisition method: Touch Imager, chemiluminescence, 0.5 s exposure.

Figure 9B p-AMPK.3
Marker Caco2 LPS+PA+NAC LPS+PA Marker

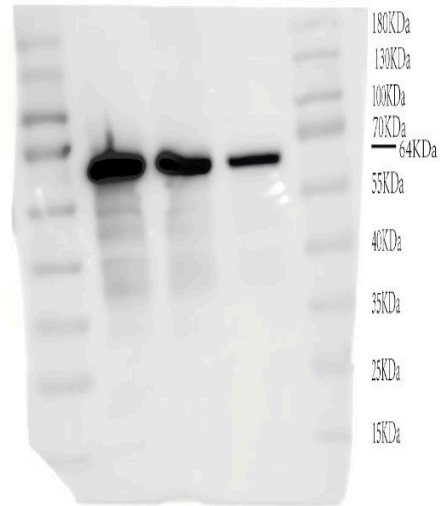

Image acquisition method: Touch Imager, chemiluminescence, 0.5 s exposure.

Figure 9B p-AMPK.3-GAPDH
Marker Caco2 LPS+PA+NAC LPS+PA Marker

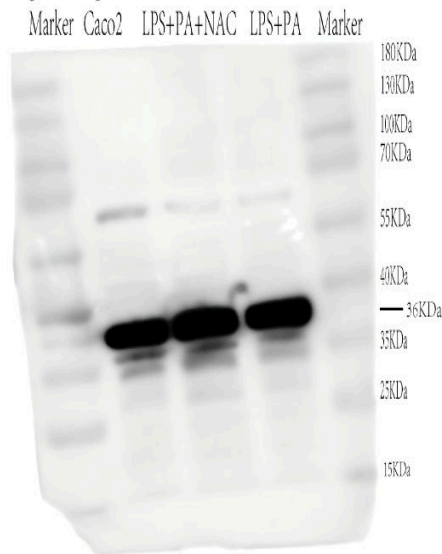

Image acquisition method: Touch Imager, chemiluminescence, 0.5 s exposure.

AMPK

Figure 9B AMPK.1
Marker Caco2 LPS+PA+NAC LPS+PA Marker

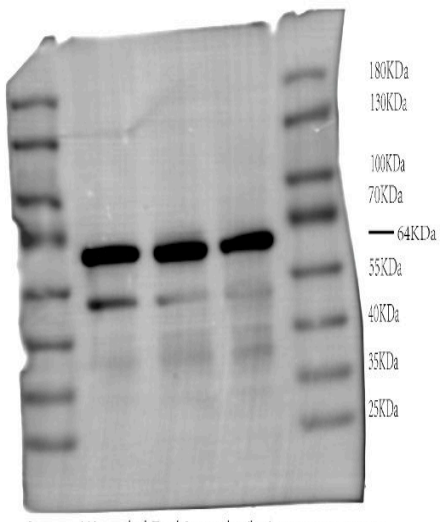

Image acquisition method: Touch Imager, chemiluminescence, 0.5 s exposure.

Figure 9B AMPK.1-GAPDH
Marker Caco2 LPS+PA+NAC LPS+PA Marker

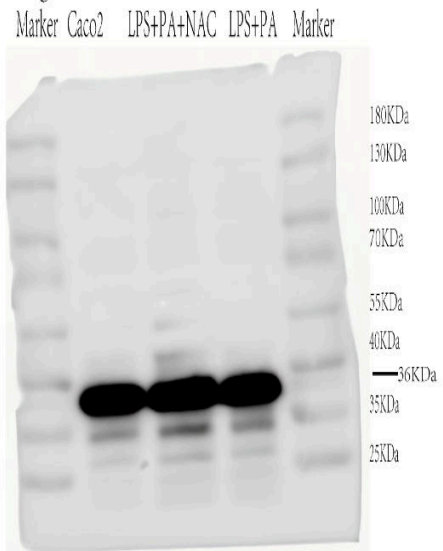

Image acquisition method: Touch Imager, chemiluminescence, 0.5 s exposure.

Figure 9B p-AMPK.2
Marker Caco2 LPS+PA+NAC LPS+PA Marker

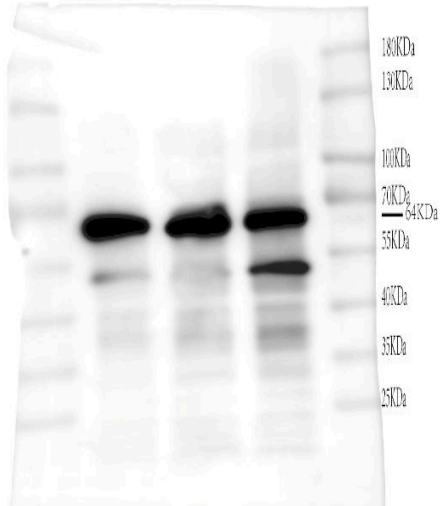

Image acquisition method: Touch Imager, chemiluminescence, 0.5 s exposure.

Figure 9B p-AMPK.2-GAPDH
Marker Caco2 LPS+PA+NAC LPS+PA Marker

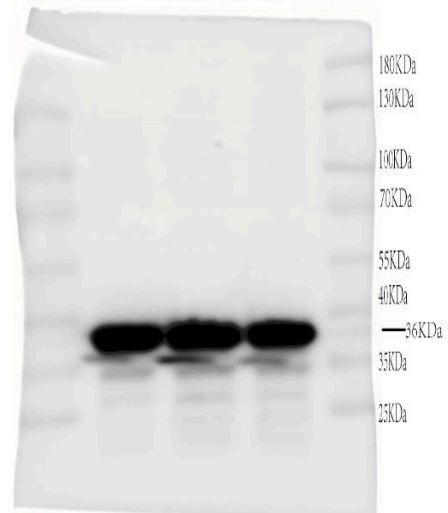

Image acquisition method: Touch Imager, chemiluminescence, 0.5 s exposure.

Figure 9B p-AMPK.3
Marker Caco2 LPS+PA+NAC LPS+PA Marker

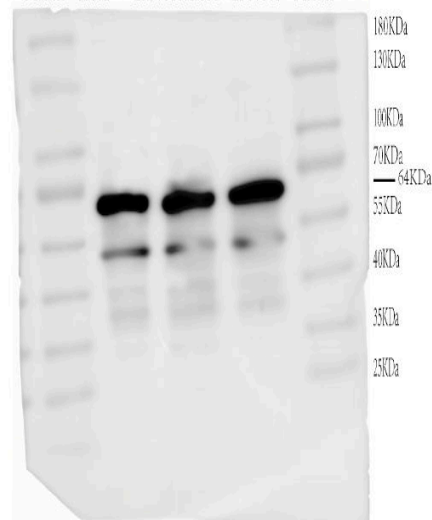

Image acquisition method: Touch Imager, chemiluminescence, 0.5 s exposure.

Figure 9B p-AMPK.3-GAPDH
Marker Caco2 LPS+PA+NAC LPS+PA Marker

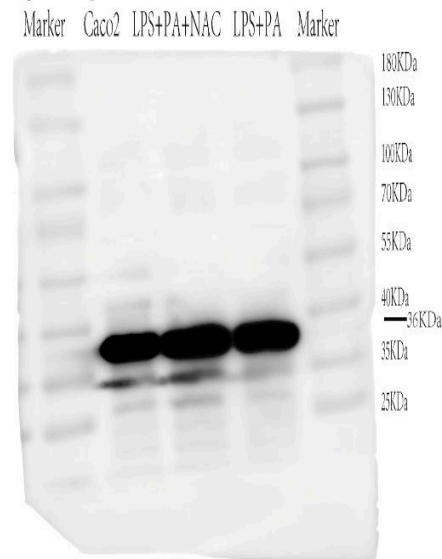

Image acquisition method: Touch Imager, chemiluminescence, 0.5 s exposure.
